# HIV Pre-Exposure Prophylaxis Interest among Female Sex Workers in Guangxi, China

**DOI:** 10.1371/journal.pone.0086200

**Published:** 2014-01-22

**Authors:** Li Ye, Suosu Wei, Yunfeng Zou, Xiaobo Yang, Abu S. Abdullah, Xiaoni Zhong, Yuhua Ruan, Xinqin Lin, Mingqiang Li, Deren Wu, Junjun Jiang, Peiyan Xie, Jiegang Huang, Bingyu Liang, Bo Zhou, Jinming Su, Hao Liang, Ailong Huang

**Affiliations:** 1 Guangxi Key Laboratory of AIDS Prevention and Treatment, School of Public Health, Guangxi Medical University, Nanning, Guangxi, China; 2 The People's Hospital of Guangxi Zhuang Autonomous Region, Nanning, Guangxi, China; 3 Department of Medicine, Boston Medical Center, Boston University School of Medicine, Boston, Massachusetts, United Staes of America; 4 School of Public Health, Chongqing Medical University, Chongqing, China; 5 State Key Laboratory for Infectious Disease Prevention and Control, Chinese Center for Disease Control and Prevention, Beijing, China; 6 Nanning Center for Disease Control and Prevention, Nanning, Guangxi, China; 7 Liuzhou Center for Disease Control and Prevention, Liuzhou, Guangxi, China; 8 Beihai Center for Disease Control and Prevention, Beihai, Guangxi, China; Vanderbilt University, United States of America

## Abstract

**Objectives:**

Acceptability of pre-exposure prophylaxis (PrEP) and willingness to participate in a clinical trial for both safety and efficacy of PrEP were investigated among female sex workers (FSWs) in Guangxi, China.

**Methods:**

A cross-sectional study was performed in three cities in Guangxi. Structured, self-administered questionnaires were used to assess the acceptability of PrEP and the willingness to participate in a clinical trial. Multivariable logistic regression models were fitted to identify predictors.

**Results:**

Among 405 participants, 15.1% had heard of PrEP. If PrEP was deemed to be effective, safe and provided for free, 85.9% reported that they would accept it, and 54.3% of those who accepted PrEP said that they would participate in a clinical trial. The increased acceptability of PrEP was associated with working in male dominated venues, higher income, a poor family relationship, better HIV/AIDS knowledge, not realizing HIV risk from unfamiliar clients, not being forced to use condoms by the gatekeepers, consistent use of condoms, and use of drugs to prevent STD infection. The increased willingness to participate in a clinical trial was associated with a poor family relationship, better HIV/AIDS knowledge, not realizing HIV risk from unfamiliar clients, a willingness to adhere to daily PreP use, and not being concerned about discrimination by others. The main reason for rejecting PrEP or participating in a clinical trial was the concern about the side effects of PrEP.

**Conclusions:**

Acceptability of PrEP among Guangxi FSWs is relatively high, indicating that PrEP intervention programs may be feasible for Chinese FSWs. Given the fact that most of the participants had never heard of PrEP before, and that family, gatekeepers, and social discrimination could significantly affect its acceptability, a comprehensive mix of multiple interventions is necessary for the successful implementation of a PrEP program among this population in Guangxi.

## Introduction

In the past two decades, China has experienced a rapid expansion of the HIV/AIDS epidemic. The Chinese Ministry of Health, the World Health Organization (WHO) and the United Nations Programme on HIV/AIDS (UNAIDS) estimated that by the end of 2011, about 780,000 people were living with HIV in China, of whom 154,000 are living with AIDS [1]. Sexual transmission continues to be the primary mode of transmission in China. The rates of heterosexual transmission have increased from 30.6% in 2006, to 47.1% in 2009, and to 52.2% in 2011 [1]. Guangxi, a southwestern province in China, has the second highest number of HIV/AIDS infections in China after Yunnan province. Guangxi shares a border with Vietnam and serves to connect China with the Association of Southeast Asian Nations (ASEAN), which is a geopolitical and economic organization of ten countries located in Southeast Asia with a population of approximately 600 million people. Trade and travel between ASEAN and China have increased in recent years, and this rapid growth and development has made Guangxi a major focus for the movement of a mobile population comprising traders, transportation workers and tourists. The prosperous economy, international contact, and increased tourism in Guangxi have created a demand and market for commercial sex. According to statistics from local Center for Disease Prevention and Control (CDC), there were at least 50,000 female sex workers (FSWs) in Guangxi in 2009, although the actual number is believed to be substantially higher [2]. In Guangxi, the transmission of HIV among the mobile population and FSWs is a growing problem. In 2011, it is estimated that 93% of new HIV/AIDS cases in Guangxi were due to unprotected sex mostly through the commercial sex trade, and the proportion of HIV-positive individuals among streetwalkers in Guangxi is several times higher than the average (∼1%) among sex workers as a whole in China (Guangxi Center for Disease Prevention and Control. unpublished data, 2012).

The use of barrier protection methods such as condoms during high-risk contact had slowed the HIV epidemic worldwide [3], [4]. A number of prevention interventions targeting FSWs in China had been implemented in response to the growing epidemic [5]. Venue-based education and condom promotion represented the typical intervention approach. Some adapted internationally validated programs such as Voluntary Counselling and Testing (VCT) and 100% Condom Use Programs (CUP) [5]. Significant intervention effects were reported in a number of studies, especially an increase in HIV/STI-related knowledge and condom use rates [5]. Condom use, however, is mainly male-controlled and the effectiveness is limited by the need for women to negotiate their use with their sexual partners [6]. Chinese FSWs, as well as Chinese women in general, who are subjected to biological, cultural, economic, judicial and political influences, are often in a relatively passive position [7]. Moreover, Chinese FSWs might be reluctant to argue with their clients, fearing both income loss and violence [8], [9], [10], [11], [12], [13]. Condom failure, including ripping and shedding, is also a recognized problem for FSWs in southern China [14]. Therefore, a female-controlled method of preventing HIV infection is urgently needed [15], [16].

Pre-exposure prophylaxis (PrEP), a potentially effective strategy for the biomedical prevention of HIV transmission, has been a focus of recent studies [17], [18], [19], [20]. In July of 2012, the Food and Drug Administration (FDA) in the United States has approved emtricitabine/tenofovir disoproxil fumarate (FTC/TDF) as PrEP to prevent HIV acquisition in adult men and women. Currently, the US remains the only country where antiretrovirals (ARVs) are licensed for use as PrEP. The rationale for the use of ARV prophylaxis is based on its efficacy in infants exposed to HIV-1 during birth and breast feeding [21], and its partial or full protection against simian or simian/human immunodeficiency virus (SIV/SHIV) challenged in primates [22], [23], [24]. Up to now, eight randomized double-blind placebo-controlled clinical trials of PrEP have been completed or are ongoing worldwide [25]. Four clinical trials including CAPRISA 004 [26], iPrEx [27], TDF2 Study [28], and the Partners PrEP study [29] demonstrated that PrEP protects against the sexual acquisition of HIV. The PrEP strategies in these clinical trials used the antiretroviral tenofovir, either as a vaginal gel or as daily oral tenofovir disoproxil fumarate, alone or coformulated with FTC. However, two trials, FEM-PrEP [30] and VOICE [25] trials failed to demonstrate HIV protection with PrEP. It appears likely that poor adherence was a major reason for why the use of oral Truvada failed to demonstrate a protective effect [31], [32]. The other two trials, Bangkok TDF Study and FACTS 001, are still ongoing [25]. The reduction in rates of HIV incidence in the four successful clinical trials varied from 39% to 75% [26], [27], [28], [29]. A sub-study of the iPrEx trial showed that a daily dose of oral PrEP could reduce the risk of HIV transmission by up to 99% if adherence to PrEP medications was sufficient [33]. Collectively, PrEP intervention has been shown to have a significant impact on populations at greatest risk of HIV acquisition.

The success of the PrEP strategy depends heavily on the user's willingness to use it. Two populations in China, men who have sex with men (MSM) and serodiscordant heterosexual couples, have been recently investigated for their willingness to use PrEP. The acceptance rate was 67.8% [34] and 84.6% [35], respectively. However, few studies to date have documented demographic, behavioural, and psychosocial characteristics of PrEP use or willingness to PrEP use in China. The study conducted among FSWs in Xinjiang, China indicated that work condition, HIV/AIDS knowledge, concerns about drug security, effectiveness and cost were associated with the willingness to use PrEP [37]. Studies abroad also reported a relatively high acceptability of PrEP in different population groups [36], [37], [38], [39] and identified several factors associated with the willingness to use PrEP, including cost [36], [37], [38], effectiveness of PrEP medication [36], [37], side effects of medication [36], [37], [38], educational attainment [38], income [38], and perceived discrimination [36]. Furthermore, in assessing the risk of HIV infection among FSWs, empirical research globally has addressed various factors including HIV and STI knowledge [40], [41], [42], [43], work condition [41], [44], income [40], [41], [42], condom use [40], [41], [42], gatekeeper support [13], [45], sexual practices [41], [46], [47], [48], STI/HIV protection methods followed [40], [41], [46], [47], [48]. Several studies reported a few structural factors such as social stigma against FSWs, poor environmental support, poor family relationship, economic deprivation, and violence as major obstacles to HIV/STI prevention in this population [14], [43], [44], [49], [50], [51], [52]. In this study, we evaluated the acceptability of PrEP and the willingness of FSWs to participate in a clinical trial in Guangxi. We analysed the factors that are associated with PrEP acceptability and willingness to participate in a clinical trial. We included multiple factors that have been identified to influence HIV prevention among FSWs in earlier studies [36]–[52] as described above, including demographic characteristics, knowledge of and attitudes towards AIDS and HIV prevention, behavioural characteristics, psychological status, sexual practices and structural factors (e.g., social services, social discrimination, family relationship, attitude of gatekeepers, influence from friends). As a feasibility study for the clinical trial, this investigation will provide evidence and information for future promotion of PrEP among FSWs in China.

## Methods

### Ethics Statement

Ethical approval of this study was obtained from the Ethics and Human Subjects Committee (EHSC) of Guangxi Medical University. All participants provided their written informed consent to participate in the study.

### Materials and Methods

Nanning, Liuzhou, and Beihai cities in Guangxi province were selected as study sites. These three cities are known as hotspots of the commercial sex trade in Guangxi province, and the HIV prevalence is high in these cities (Guangxi Center for Disease Prevention and Control. unpublished data, 2012).

### Participants

In this study, “sex work” refers to “direct prostitution”, namely, the sexual services with the primary purpose for money [53]. The participants were recruited from either male dominated venues (hotels, nightclubs and massage parlours) or street-based places (barber shops and streetwalkers) [53]. Between July 2009 and April 2010, 405 FSWs from Nanning (150), Liuzhou (160), and Beihai (95) cities in Guangxi province were recruited via a combination of convenience sampling with the assistance of local social and health-care institutions that serve FSWs in the areas and snowball sampling for participants who met study criteria. Women aged 18 years and above who understood and could speak either Mandarin or the local dialect, had self-reported negative or unknown HIV seropositivity, and had been engaged in sex work for more than one month were eligible for the study. When an eligible FSW was identified, the researchers or research assistants (RAs) would provide her an informed consent (Documents S1 and S2), which included a brief introduction to PrEP, the purpose and procedures of the research, an overview of the risks and benefits of participation, as well as the methods used to protect personal privacy, and invited her to participate. Those who could not understand the content of the informed consent and could not provide written informed consent were excluded. All participants were allowed to use a nickname or a pseudonym, and they received a modest reimbursement (40 Chinese Yuan, equivalent to ∼ U.S. $6.5) for their participation.

### Procedures

Before the formal investigation, we conducted two small pilot investigations (with 20 participants in each pilot) to improve participant understanding of the educational materials and questionnaire. For each survey, two experienced RAs (lecturers or MD/Ph.D. students at Guangxi Medical University) interviewed the participants and collected data. The RAs were trained in the research methods and PrEP-related knowledge beforehand. Upon recruitment, the RAs introduced the aim of this study and the content of informed consent either to an individual FSW or to a group. The introduction was conducted in either Mandarin or the local dialect at a private location convenient for the participants. The explanation of PrEP is as follows:

“PrEP is the strategy of using antiretroviral agents (that is a form of medication effective for treating HIV/AIDS) to prevent HIV infection in HIV-negative persons, which is one of the newer preventive strategies being evaluated in research studies globally. This strategy has evolved from the studies in monkeys that have demonstrated a reduced risk of infection from simian immunodeficiency virus in animals that were pretreated with antiretroviral (ARV) agents. Recent studies on humans also reported that women who received PrEP were three times less likely to acquire HIV infection than women who received a placebo, which is inactive medication. Several clinical trials using new medications, namely, tenofovir disoproxil fumarate (TDF) and/or emtricitabine (FTC) for PrEP are ongoing or have been completed in some countries. There are two suggested regimens and doses so far, which were recommended for clinical trails of PrEP. One is TDF, whose suggested oral dose is TDF 300 mg once a day; the other is two-drug PrEP (TDF/FTC), and the suggested oral dose is TDF/FTC 300 mg/200 mg once a day. Both drugs have possible side effects, such as nausea, vomiting, headache etc, similar to other ARV drugs (that is the drugs used to treat HIV/AIDS), but this should improve after a few weeks of PrEP use. Unlike ARV drug treatment for AIDS patients, however, treatment withdrawal and resistance prevention should be considered simultaneously. The optimal PrEP regimen is not known so far for PrEP clinical research. Although there remain some challenges in PrEP research, it is still a promising strategy for HIV prevention. In this study, we want to know, if PrEP is safe and effective. Do you want to participate or do you have still have concerns about this study?”

### Questionnaire

After obtaining the participant's verbal consent that they had fully understood what PrEP is and after signing the informed consent, the RAs provided each participant with a self-administered questionnaire (Documents S3 and S4). During the process of questionnaire completion, communication between the RAs and the participants continued. The participants were encouraged to ask the RAs to explain any content that was unclear to them. In addition, terms in the questionnaire that were not understood by participants were further explained in plain language by the RAs. The questionnaire is comprised of 3 parts as follows: (**1**) **Demographic characteristics**. Twelve items were investigated to show the socioeconomic characteristics of FSWs, including ethnicity, age, birthplace, household registration, residence status, education level, work environment, monthly income, marital status, whether they have children, family relationships, and the attitudes towards their own health; (**2**) **Knowledge of AIDS, attitude towards AIDS, the situation and access to AIDS prevention services, and history of AIDS-related behaviour.** Thirteen questions were asked to assess knowledge of AIDS (Cronbach's alpha  = 0.84), which were adapted from the HIV sentinel surveillance questionnaire from the Chinese Center for Disease Control and Prevention (e.g., “Does AIDS spread through needle sharing?”; “Does AIDS spread through mosquito bites?”, etc). Thirteen questions were asked to assess their attitudes towards AIDS (e.g., “What's your attitude towards people infected with HIV?”; “Do you consider there is a risk of HIV from unfamiliar clients?”). Eleven questions were asked to demonstrate whether the participants received or their willingness to receive any services regarding AIDS prevention from government departments or social organizations (e.g., “In the last six months, have you received any services for AIDS prevention?”; “In last 6 months, had you ever had an HIV test?”; “Would you like to have a free HIV test if you haven't had one before?”; “How would you like to learn more about AIDS?”, etc). We also included 43 items to understand the behavioural characteristics of the participants and the external factors that influence their behaviour (e.g., “How old were you when you first had sex?”; “How often did you use condoms when having sex with clients in the past six months?”; “Have you ever used drugs to prevent STD infection?”; “Have you ever had the following symptoms of venereal diseases?”; “What's the attitude of the gatekeepers toward using condoms?”; “Did the gatekeeper force you to use condoms when you had sex with clients?”). (**3**) **Knowledge, attitude and willingness of HIV-prevention methods.** This section covers awareness of, use of and concerns about PrEP and condom use; the willingness to use PrEP or to participate in a clinical trial in term of cost, adherence, and accessibility; and perceived behavioral changes after PrEP. We asked 43 questions to investigate the above information. Acceptability of PrEP use was investigated through the question, “If PrEP were safe and effective, how likely would you be willing to use it?” Willingness to participate in a clinical trial was investigated through the question, “If PrEP were safe and effective, how likely would you be willing to participate in a clinical trial?” Participants were asked to report their intention on a 5-point scale: 1. absolutely willing; 2. probably willing; 3. unknown; 4. probably unwilling; 5. absolutely unwilling. Data were dichotomized into “willing to use or participate” (score of 1 and 2) and “unwilling to use or participate” (3 or higher).

### Data analysis

Questionnaire data were double-checked and entered into EpiData software (EpiData 3.0 for Windows, EpiData Association, Odense, Denmark). The data were then converted and analysed using SPSS for Windows Version 15.0 (SPSS, Chicago, IL, USA). Univariate analyses including chi-squared test and Fisher's exact test were performed to evaluate the associations of acceptability of PrEP use or willingness to participate in a clinical trial with other variables. Variables significant at a level of *p≤*0.10 were fitted in a multivariable logistic regression model to estimate the factors associated with acceptability of PrEP use or willingness to participate in a clinical trial, and only the factors significant at a level of 0.05 (two-tailed) were reported.

The data, in the form of computer electronic documents and paper materials were deposited in Guangxi Key Laboratory of AIDS Prevention and Treatment, School of Public Health, Guangxi Medical University. The data were shared by Guangxi Medical University and all cooperating institutions (see the institutions of all authors of this paper). With the agreement of Guangxi Medical University and all cooperating institutions of this study, the data will be available to other individuals and institutions.

## Results

### Demographic characteristics of participants

A total of 405 FSWs completed the self-administered questionnaire. Table 1 shows the demographic characteristics of the participants. Of the respondents (n = 405), 37.0% were from Nanning, 39.5% from Liuzhou, and 23.5% from Beihai city, respectively. The majority of participants (62.2%) were from male dominated venues (hotels, nightclubs and massage parlours), and the rest from street-based places (barber shops and streetwalkers). Participants ranged in age from 18 to 53 years (median  = 28.0, mean  = 29.1, SD = 7.7). In total, 26.4% of participants came from urban areas, 73.6% from rural areas; 60.7% belonged to the Han ethnic group, 39.3% were ethnic minorities; 24.2% had primary school education level, 60.2% had junior high school education level, and 15.6% had senior high school or above education. Of all participants, 16.8% reported a monthly income of <1,000 RMB (Chinese Yuan, equivalent to ∼ U.S. $161), 57.5% between 1,000 RMB and 3,000 RMB, 25.7%>3,000 RMB (equivalent to ∼ U.S. $482); 20.5% of participants reported never having been married, 68.4% were married or cohabitated, 11.1% were divorced, separated or widowed. On average, the FSWs had had eight sexual partners in the past seven days (median  = 7.0, mean  = 8.5, SD  = 6.0), in a range of 1 to 50.

**Table 1:** Demographic characteristics of participants.

Characteristics	Total (N)	Percent (%)
Total	405	100
Cities (Location)		
Nanning	150	37
Liuzhou	160	39.5
Beihai	95	23.5
Age		
18–24	135	33.3
25–29	103	25.4
30–34	74	18.3
35–39	43	10.6
over 40	50	12.4
Ethnicity		
Han	246	60.7
Zhuang	125	30.9
Others	34	8.4
Registered residency		
Rural	107	26.4
Urban	298	73.6
Education level		
Primary school	98	24.2
Junior high school	244	60.2
Senior high school or above	63	15.6
Marital status		
Unmarried	83	20.5
Cohabiting	124	30.6
Married	153	37.8
Others (divorced or widowed)	45	11.1
Having children		
Yes	177	43.7
No	228	56.3
The relationship with family		
Good	301	74.3
General	87	21.5
Bad	17	4.2
Income per month		
≤1000 RMB	68	16.8
1001–3000 RMB	233	57.5
>3000 RMB	104	25.7
Work conditions		
Male dominated venues (hotels, nightclubs and massage parlours)	252	62.2
Street-based places (barber shops and streetwalkers)	153	37.8

### Self-reported AIDS/STI knowledge, AIDS/STI history, and attitude towards AIDS/STI

Among the 405 FSWs, only 26.1% self-reported having a good HIV/AIDS knowledge, 36.8% believed that it is hard to prevent HIV infection; 92.8% worried about contracting HIV, 74.8% reported consistent use of condoms, and 66.4% of participants had had an HIV test; 50.4% of the FSWs surveyed reported at least one STI symptom in the last six months, and 13.3% participants had ever been diagnosed with an STI.

### Acceptability of PrEP use or willingness to participate in a clinical trial

Of all participants, 15.1% had heard of PrEP; 85.9% participants reported that they were willing to use PrEP in the future if it was proven to be safe and effective (Table 2). Of those unwilling to accept PrEP (57), the majority (89.5%) were concerned about the side effects of PrEP, 50.9% thought they were not at risk of HIV through commercial sex (Table 3). Other reasons included the belief that PrEP was not necessary or not effective (36.8%), concern about objections from family (31.6%) and discrimination by others (17.5%) (Table 3). Of the 348 participants who were willing to accept PrEP, 14.9% had heard of PrEP, 54.3% indicated that they would participate in a clinical trial. Of those unwilling to participate, the majority (81.8%) were concerned about the side effects of PrEP, followed by PrEP not being necessary or not being effective (42.8%), self-perceived no HIV risk (27.7%), concern about objections from family (20.7%), and discrimination by others (11.3%) (Table 3).

**Table 2:** Willingness to accept PrEP or participate in a clinical trial (N, %).

	Total	Having heard of PrEP	Willing	Unwilling
Willingness to accept PrEP	405	61 (15.1)	348 (85.9)	57 (14.1)
Willingness to participate in a clinical trial	348	52 (14.9)	189 (54.3)	159 (45.7)

**Table 3:** Reasons for refusing PrEP or participating in a clinical trial.

Reasons	Reasons for refusing PrEP (N, %)	Reasons for refusing a clinical trial (N, %)
Total	57 (100)	159 (100)
Concern about side effects	51 (89.5)	130 (81.8)
Self-perceived no HIV risk from commercial sexual behaviours	29 (50.9)	44 (27.7)
Not necessary or not effective	21 (36.8)	68 (42.8)
Concern about objections from family	18 (31.6)	33 (20.7)
Concern about discrimination by others	10 (17.5)	18 (11.3)

### Factors associated with HIV PrEP acceptability

Results of univariate analysis of factors associated with HIV PrEP acceptability indicated that statistically significant (*p*≤0.10) variables included location, work conditions, monthly income, family relationships, HIV/AIDS knowledge, weekly client number, not having had oral sex in the last six months, whether the gatekeepers forced FSWs to use condoms, whether they consistently used condoms, whether they realized the risk of HIV from unfamiliar clients, whether they ever used drugs to prevent STD infection (data not shown). These 11 significant variables were entered in a multivariable logistic regression model, and only those factors significant at *p*<0.05 were shown in the final model (Table 4). The analysis showed that eight variables were associated with PrEP acceptability. An increased acceptability was associated with working in male dominated venues (hotels, nightclubs and massage parlours), higher monthly income, poor family relationships, better HIV/AIDS knowledge, not realizing HIV risk from unfamiliar clients, not being forced by the gatekeepers to use condoms, consistent use of condoms, and the use of drugs to prevent STD infections (Table 4).

**Table 4:** Logistic regression analysis of willingness to use PrEP.

Factors	Control	β	*SE*	Waldχ^2^	*P*	OR	95%CI
Work environments							
Male dominated venues	Street-based places	−0.814	0.312	6.791	0.009	2.257	1.223∼4.167
Monthly income							
≤3000Yuan	>3000Yuan	1.36	0.47	8.371	0.004	0.257	0.102∼0.645
Family relationship							
Good	Bad	1.083	0.431	6.326	0.012	0.338	0.146∼0.787
HIV/AIDS knowledge							
Good	Bad	−1.03	0.469	4.818	0.028	2.802	1.117∼7.033
Whether the gatekeepers forced FSWs to use condoms							
Yes	No	0.84	0.33	6.486	0.011	0.432	0.226∼0.824
Whether consistently used condoms with clients							
Yes	No	−1.102	0.358	9.453	0.002	3.01	1.491∼6.076
Realizing unfamiliar clients as being an HIV risk							
Yes	No	1.012	0.516	3.842	0.05	0.363	0.132∼1.000
Have used drugs to prevent STD infections							
Yes	No	−1.272	0.646	3.882	0.049	3.57	1.107∼12.659

### Factors associated with a willingness to participate in a clinical trial

Results of univariate analysis of factors associated with the intention to participate in a clinical trial indicated that statistically significant (*p*≤0.10) variables included location, work conditions, having children, family relationships, HIV/AIDS knowledge, having STD symptoms in the last six months, not realizing HIV risk from unfamiliar clients, considering temporary sexual partners as being an HIV risk, clients' attitude on PrEP use, their attitude on taking medicine every day, considering that they were able to protect themselves against HIV infection, concern about discrimination by others, whether the gatekeepers forced FSWs to use condoms (data not shown). These 13 significant variables were entered in a multivariable logistic regression model, and only those factors significant at *p*<0.05 were shown in the final model (Table 5). The analysis showed that five variables were associated with the willingness to participate in a clinical trial. An increased willingness was associated with a poor family relationship, better HIV/AIDS knowledge, not realizing HIV risk from unfamiliar clients, willingness to adhere to a daily medication, and not being worried about discrimination by others (Table 5).

**Table 5:** Logistic regression analysis of intention to participate in a clinical trial.

Factors	Control	β	*SE*	Waldχ^2^	*P*	OR	95%CI
Family relationship							
Good	Bad	−0.705	0.285	6.117	0.013	0.494	0.283∼0.864
HIV/AIDS knowledge							
Good	Bad	0.855	0.301	8.083	0.004	2.35	1.304∼4.236
Realizing unfamiliar clients as being an HIV risk							
Yes	No	−0.633	0.316	4.022	0.045	0.531	0.286∼0.986
Can they adhere to taking medicine every day							
Yes	No	1.186	0.267	19.747	0	3.272	1.94∼5.52
Concern about discrimination by others							
Yes	No	−1.279	0.264	23.508	0	0.278	0.161∼0.414

## Discussion

Increasing evidence has confirmed the safety and efficacy of PrEP in preventing HIV-1 in certain populations [54]. Our study showed that only 15.4% FSWs in Guangxi had ever heard of PrEP. Nevertheless, among this diverse population, the majority (85.9%) indicated an interest in using PrEP after learning about its efficacy and safety. The rate was higher than that of the MSM population either in China (67.8%) [34] or in the United States (67%,−74.4%) [38], [55], and was comparable to that of serodiscordant heterosexual couples in China (84.6%) [35]. The high level of PrEP acceptability among Chinese FSWs indicated that PrEP intervention programmes may be feasible among Chinese FSWs in the future. In our study, 74.8% of the participants reported consistent use of condoms, and 66.4% of participants had had an HIV test, reflecting the significant effects of prevention interventions (venue-based education and condom promotion) targeting high-HIV-risk populations, including FSWs, implemented in recent years in China [5]. However, compared with condom use, PrEP is a female-controlled method that can be used by women without the consent of their sexual partners. Given that Chinese women are often in a passive position in sexual activity, PrEP might be a particularly appropriate and effective HIV-prevention option for Chinese women, especially FSWs.

Previous studies have reported a relatively high acceptability of PrEP in different populations across the world [36], [37], [38], [39] and identified some factors affecting the willingness to use PrEP, including expense [36], [37], [38], effectiveness [36], [37], side effects [36], [37], [38], education [38], moderate income [38], and discrimination [36]. Our study identified eight predictors of PrEP acceptability among FSWs in Guangxi, including work environment, monthly income, family relationship, HIV/AIDS knowledge, not realizing HIV risk from unfamiliar clients, not being forced to use condoms by the gatekeepers, consistent use of condoms, and use of drugs to prevent STD infections (Table 4). Among them, consistent with previous studies, higher income, better HIV/AIDS knowledge, better work conditions and consistent use of condoms were found to be important for PrEP acceptability [36], [37], [38], [44], [56]. However, other factors, including family relationships and being forced to use condoms by gatekeepers which are shown to be important for PrEP acceptability in our study are different from previous results [36], [37], [38]. We found that poor family relationships are associated with increased PrEP acceptability, which is contrary to our original expectations. We believe that a good family relationship would contribute to PrEP acceptability, as some studies have shown that intimate and/or marital relationships were integral to willingness or adherence to PrEP use [52]. Future studies should explore this issue with more details. In this study, we also found the FSWs working in establishments that ordered the use of condoms were less likely to accept PrEP, probably due to the fact that they believed the protective effect of condoms is enough or because they were afraid – due to cost issues or the need for a complicated process – of being coerced into PrEP use. On the other hand, this reflects the role of gatekeepers in FSWs acceptance of PrEP. If the gatekeepers understand the importance of PrEP in the prevention of HIV-1, their subsequent suggestion that PrEP should be used may increase FSWs acceptance of PrEP. Client types were also a factor to influence PrEP acceptability. We found that realizing HIV risk from unfamiliar clients was associated with low PrEP acceptability. One possible reason for this is the FSWs' concern about the efficacy of PrEP and they prefer using condoms to prevent the greater threat of HIV infection from unfamiliar clients whose HIV status is unknown. In our study we did not find that the FSWs who accepted PrEP would likely decrease concurrent condom use, which also reflects their concern about the efficacy of PrEP. Similar results can be seen in one previous study, which showed that PrEP could be acceptable and beneficial even with the consistent use of condoms [57]. Despite all this, following the US CDC statement [58], we want to emphasize that PrEP should not be considered as the first line preventive measure against HIV. Instead, it should be a supplement to other effective preventive measures, such as condom use. Although PrEP has been shown to be effective in four clinical trials [26], [27], [28], [29], it has failed to demonstrate HIV protection in two other trials [25], [30]. Low adherence to consistent use of PrEP is the leading hypothesis to account for the lack of efficacy [31], [32]. Importantly, one study showed that those who expressed a willingness to accept PrEP would be likely to decrease condom use [59]. These results showed that a competitive selection between PrEP and condom use might take place due to people being more inclined to use a simple and effective way to protect themselves from HIV infection. Taken together, a combination of PrEP with other effective strategies, including consistent condom use, should be seriously considered when PrEP is introduced.

To achieve HIV/STD prevention goals, a model program should intervene at multiple levels: structural or environmental, community, social network, and individual [60]. Given the sensitive nature of FSWs in China, working at each of these levels would need strategies involving multiple stakeholders. However, building empowerment among FSWs in China should be a priority to ensure their and their families' wellbeing. Organizations that are working to support FSWs in China could work with relevant stakeholders or authorities to adopt empowerment strategies [60]. Kar et al [61] suggested an approach they called EMPOWER: Education and leadership development, Media use and advocacy, Public education and participation, Organizing associations and unions, Work training and micro-enterprise, Enabling services and assistance, and Rights protection and promotion. The EMPOWER strategies restructure risk environments to enable or “empower” sex workers to protect their health and families' well-being [62]. However, public health workforce should work carefully as legalization of female sex work is a sensitive and complicated topic in China.

Although the acceptance rate is relatively high among the FSW population in China, only half (54.3%) of those who accepted PrEP indicated that they would participate in a clinical trial. The willingness to participate in a clinical trial among FSWs in Guangxi is slightly lower than that in Xinjiang reported by a recent study (69.3%) [37]. We found that among the FSWs in Guangxi, willingness to participate in a clinical trial to evaluate PrEP was associated with poor family relationships, better HIV/AIDS knowledge, not realizing HIV risk from unfamiliar clients, being able to adhere to taking medicine on a daily basis, and not being worried about discrimination by others. The FSWs who have a better knowledge of HIV/AIDS and who would adhere to taking their PrEP medicine every day are more inclined to participate in a clinical trial, indicating that better HIV/AIDS knowledge and willingness to adhere to daily PreP use are two important predictors of candidates inclined to participate in PrEP clinical trials. This may be due to the fact that the FSWs who had better HIV/AIDS knowledge and adherence have a stronger self-protective awareness, which encourages them to seek more protective measures. Similar results were reported in another recent study [37]. This also implies that one effective way to increase the willingness of FSWs to participate in a clinical trial is strengthening the dissemination of HIV/AIDS knowledge in China. This investigation also found that the FSWs who had bad family relationships and were concerned about discrimination by others are more likely to reject participation in a clinical trial. These results suggest that joint intervention and reducing discrimination may be effective methods to improve participation rates of Guangxi FSWs in PrEP clinical trials.

The main reason for rejecting either use of PrEP or participation in a clinical trial is the concern about the side effects of PrEP (Table 3), even though several trials have demonstrated high safety of PrEP among South African women, MSM, heterosexuals, and East African HIV serodiscordant couples [20], [26], [27]. The availability of longer-term safety data and safety profiles in pregnant and breastfeeding women may have a significant effect on PrEP acceptance. The levels of HIV/AIDS and PrEP knowledge are also an important factor for the rejection of PrEP use or participation in a clinical trial (Table 3), indicating the importance of HIV/AIDS and PrEP education as a key component of PrEP roll-out in China. In addition to the reasons above, social pressures, including the participants' concerns regarding family objection and discrimination by others, play a role in their refusal to accept PrEP or to participate in a clinical trial (Table 3). However, social pressures are only partly to blame for the refusal to accept PrEP or participate in a clinical trial (11.3%–31.6%), potentially indicating the effects of society-based HIV/AIDS education programmes implemented in recent years in China [5]. Nevertheless, social pressures, especially discrimination in relation to PrEP use, are still an equally important factor for the future of PrEP implementation in China, as previous studies have shown [36], [63].

This study has some limitations. Firstly, we investigated attitudes and behaviours based on interviews, which may be limited by social desirability bias and lead to overestimation of the acceptability of PrEP. Secondly, participants were assessed on the likelihood of a hypothetical PrEP; therefore, it is inevitable that some questions were answered subjectively. Thirdly, non-probability sampling method was restricted in the inferences of the population. Sex work is still illegal in China. Fear of police crackdowns and arrest leads to Chinese FSWs existing as a “hidden” population in society. A considerable proportion of FSWs refused to participate in the investigation because they didn't want their name or profession to be known by others. Thus, random sampling, time location sampling (TLS) or respondent driven sampling (RDS) are not practical at this time for a study of the FSW population in China. The convenience sampling and snowball sampling used in our study might have led to selection bias and limit the generalizability of our research findings. Also, we did not know the exact number of FSWs who refused to participate in the investigation because snowball sampling was used in our study. Finally, we did not investigate the possibility that participants may want to use PrEP, but not in the context of a clinical trial, which to some degree, leads to a lack of connection between the two investigations.

## Conclusions

Our study found that the acceptability of PrEP is high among FSWs in Guangxi; however, only half of those willing to accept PrEP intended to participate in a clinical trial to evaluate the effectiveness of PrEP. The main factors influencing the acceptability of PrEP include HIV/AIDS knowledge, income, consistent use of condoms, and the use of drugs to prevent STD infections. The main factors influencing the willingness to participate in a clinical trial include HIV/AIDS knowledge and their attitude towards taking medicine every day. The main reason for rejecting PrEP use or participation in a clinical trial was the concern about the side effects of PrEP. In addition, the impact from family, gatekeepers, and social discrimination could significantly affect the willingness of FSWs to accept PrEP or to participate in a clinical trial. These results indicate that a comprehensive mix of multiple interventions – not just focusing on FSWs, but also on their family, gatekeepers, and public opinion – is necessary for the successful implementation of a PrEP programme among the FSW population in Guangxi and other parts of China.

## Supporting Information

Document S1 (DOC)

Document S2 (DOC)

Document S3 (DOC)

Document S4 (DOC)
