# Supplementary material for: HIV Pre-Exposure Prophylaxis Interest among Female Sex Workers in Guangxi, China
Source: PLoS One. 2014 Jan 22;9(1):e86200. doi: 10.1371/journal.pone.0086200 (PMC3899205; doi:10.1371/journal.pone.0086200)
Supplement: Document S1 — (DOC) [file pone.0086200.s001.doc]

**Informed Consent form**

**The feasibility of PrEP implementation among high-risk populations to reduce new HIV infections in western China**

**Statement**

To investigate the awareness and acceptability of pre-exposure prophylaxis (PrEP) among female sex workers (FSWs) and potential predicting factors in Guangxi, China, we invite you to participate in key state science and technology project, *the feasibility of PrEP implementation among high-risk populations to reduce new HIV infections in western China*. This investigation will be conducted in Nanning, Liuzhou, Beihai cities in Guangxi. Heads of the investigation are Prof. Hao Liang (Guangxi Medical University, School of Public Health) and Prof. Ailong Huang (Chongqing Medical University).

**PrEP Introduction**

PrEP is the strategy of using antiretroviral agents to prevent HIV infection in HIV-negative persons, which is one of the newer preventive strategies being evaluated in randomized controlled trials globally. This strategy has evolved from the studies of non-human primates that have demonstrated a reduced risk of infection from simian immunodeficiency virus in animals that were pretreated with antiretroviral (ARV) agents. Recent studies on humans also reported that women who received PrEP were three times less likely to acquire HIV infection than women who received a placebo. Several clinical trials using tenofovir disoproxil fumarate (TDF) and/or emtricitabine (FTC) for PrEP are ongoing or have been completed in some countries. There are two suggested regimens and doses so far, which were recommended for clinical trails of PrEP. One is one-drug TDF), whose suggested oral dose is TDF 300 mg once a day; the other is two-drug PrEP (TDF/FTC), and the suggested oral dose is TDF/FTC 300 mg/200 mg once a day. Both drugs have possible side effects, such as nausea, vomiting, headache etc, similar to other ARV drugs, but this should improve after a few weeks of PrEP use. Unlike ARV drug treatment for AIDS patients, however, treatment withdrawal and resistance prevention should be considered simultaneously. No definite strategies are available so far for PrEP clinical research. Although there remain some challenges in PrEP research, it is still a promising strategy in HIV prevention. In this study, we want to know whether, if PrEP is proved to be safe and effective, you will accept it or whether you will still have worries about it.

**Your Participation**

Investigation requires you to provide some information and answers to a questionnaire. It will take you about 30 minutes to answer the questions mainly about knowledge and attitude of HIV/AIDS, your behavior, awareness of HIV/AIDS prevention, willingness to accept PrEP, and willingness to participate in PrEP clinical trails, and so on.

**Voluntary Participation**

Your participation in this research is entirely voluntary. It is your choice whether to participate or not. You may change your mind later and stop participating even if you agreed earlier. When you decide to quit this investigation, we will remove your information in this investigation, unless you agree to our reservation. In addition, during the investigation, you can refuse to answer any question you don't want to answer.

**Confidentiality and Security**

To keep participant information confidential, actual names were not required. Nicknames are permitted when signing the informed consent forms. Your information, in the form of computer electronic documents and paper materials, will be placed in centralized custody, and security setting will be configured to these documents and materials.

**Your Benefit**

You may not get other direct benefit from this investigation. However, you may learn about HIV/AIDS-related knowledge from investigators.

**Reimbursements**

You will receive a reimbursement valued at 40 Chinese Yuan (equivalent to ~ U.S. $6.5) for your participation.

**Your risk**

This investigation will not bring you any physical risk. However, due to the sensitivity of the HIV/AIDS topics, some topics may make you feel embarrassed or uncomfortable. Also, you are likely to be seen to participate in this investigation by other people and they may know your current occupation.

If your participation brings mental pressure or any other problems, or you want to learn about more information on HIV/AIDS and PrEP, you can contact with staff of our research team.

Contact information is as follows:

*Address: 22 Shuangyong Road, Guangxi Medical University, School of Public Health, Nanning city, Guangxi 530021, China.*

*Contact Person: Sousu Wei*

*Tel: 86-771-5358145*

Name or Participant Date：

Name of Certifier Date

Name of Investigator Date:
