# Supplementary material for: HIV Pre-Exposure Prophylaxis Interest among Female Sex Workers in Guangxi, China
Source: PLoS One. 2014 Jan 22;9(1):e86200. doi: 10.1371/journal.pone.0086200 (PMC3899205; doi:10.1371/journal.pone.0086200)
Supplement: Document S2 — (DOC) [file pone.0086200.s002.doc]

### 知情同意书

**我国西部高危人群暴露前用药减少HIV新发感染的可行性研究**

**知情同意书**

**研究声明**

为了了解广西女性性工作者对暴露前用药（PrEP）预防HIV感染的知晓率和可接受性以及影响可接受性的相关因素，我们邀请您参加国家重大科技专项“我国西部高危人群暴露前用药减少HIV新发感染的可行性研究”课题。本研究将在广西南宁、柳州、北海三个地区进行。主要负责人为广西医科大学公共卫生学院梁浩教授和重庆医科大学黄爱龙教授。

**PrEP简介**

PrEP是指有高感染风险的HIV 阴性个体在潜在的HIV 暴露之前或暴露期间规律定时服用抗艾滋病病毒药物(ARVs) 以期降低HIV 感染的风险。目前，全球正在进行多项随机双盲临床试验来评估PrEP的安全性和有效性。这项研究起源于对非灵长类动物（如猕猴）暴露前服用ARVs药物来降低猴免疫缺陷病毒感染的研究。研究结果显示，使用PrEP组的妇女的HIV感染率是使用安慰剂组的三分之一。几项单独使用替诺福韦（TDF）或使用替诺福韦联合恩曲他滨（FTC）的随机对照临床试验正在国外进行，部分国家已完成试验。目前推荐的服药方式有两种，一种为口服TDF（300mg），1次/天，另一种为联合用药，即口服TDF（300mg）/FTC（200mg），1次/天。两种服药方式都可能会有一定的不良反应，例如恶心、呕吐、头痛等，但服药一两个星期后会有所好转。与治疗艾滋病的药物不同，使用PrEP时应考虑出现停药和耐药的风险。到目前为止，临床试验还没有探索出一个明确的、行之有效的PrEP综合策略。虽然PrEP还面临着一些挑战，但是PrEP仍然是当前处于临床试验的最有前景的HIV预防策略之一。通过本次研究，我们想了解，假设PrEP是安全的和有效的，您是否愿意使用，以及您对PrEP的期望。

**您的参与**

研究过程需要您提供一些信息，大约花费您30分钟时间，主要询问对艾滋病的知识、态度、行为以及对艾滋病预防用药的认识、态度和接受意愿等。

**自愿参与**

参加本研究完全是自愿的。您随时可以终止参与本研究，我们将尊重您的决定。当您决定退出本研究时，我们将去除您在本研究中的信息，除非您同意我们保留。另外，在调查过程中，您可以拒绝回答自己不愿意回答的问题。

**保密原则**

研究人员会尽力对您的个人资料保密。研究过程中，我们采取了相应措施，以保证除了调查员、资料管理负责人外的其他人员不了解您的姓名和个人信息。

从您这里获得的研究数据，以计算机电子文件、录音与纸质材料的形式集中存放，并依据各类材料特征设置相应的保密措施。

退出/被排除出研究

您可以在任何时候终止参与本研究，这不会影响您任何其他权利。退出研究时，您可以要求销毁或去除您已经提供的资料。

**参与研究可能的利益**

您可能不会从参与研究中得到直接的利益，您可能从调查人员处了解到艾滋病感染及预防等相关知识。

**补偿**

您参与本研究不需要花费任何费用，完成本调查者，我们会为您提供40元现金，作为您参加研究花费时间及往返交通费的补偿，调查结束后，调查人员将立即给您现金。

**参与研究可能的风险**

本研究不会给您带来任何身体上的风险。然而，由于艾滋病话题的敏感性，某些话题可能令您感到尴尬或不适。您有可能被其他人员看见而出现谣言，他们可能伤害您或躲避您，也可能发生目前无法预测的风险。

如果您因为参与本研究而出现精神压力或任何其他问题，或者您想了解更多的HIV/AIDS和PrEP的知识和信息，您可以与研究单位有关人员联系。联系方式如下。

地址:广西南宁市双拥路22号 广西医科大学公共卫生学院；邮编: 530021；

联系人:韦所苏

联系电话:0771—5358145

研究对象姓名或指印（字迹清晰）： 签署日期：

证明人姓名（字迹清晰）： 签署日期：

调查人姓名： 签署日期：
