# Supplementary material for: HIV Pre-Exposure Prophylaxis Interest among Female Sex Workers in Guangxi, China
Source: PLoS One. 2014 Jan 22;9(1):e86200. doi: 10.1371/journal.pone.0086200 (PMC3899205; doi:10.1371/journal.pone.0086200)
Supplement: Document S3 — (DOC) [file pone.0086200.s003.doc]

**Quantitative Questionnaires**

Introduction:

Hello! We are the investigators of major national research project-feasibility of pre-exposure prophylaxis (PrEP) against human immunodeficiency virus. This investigation is mainly to investigate your awareness of health knowledge, attitude and behavior. It may involve some of your privacy, but the content of your answers will be kept strictly confidential. We only use these questionnaires to do population analyses, not to do personal analyses. We won't disclose your information to any other individuals or organizations. So we hope you answer the following questions truthfully. This investigation will take you some time. We have to say sorry for any convenience caused. Thank you for your support.

**Questionnaire based information**

Questionnaire number: **□□□□□□□□□□**(administrative division GB code + investigation object category + investigation object number)

Location: ___________ province (municipality) ___________city/district/county

Site: ____________

Informed consent: _______________ (Yes/No)

Investigator: ____________

Investigation date: __________________

**Table The questionnaire** **quality control table**

| Item | Completion situation | | Note |
| --- | --- | --- | --- |
|  | Yes | No |  |
| Introduction, clear |  |  |  |
| All questions were answered, no leakage |  |  |  |
| No logical errors |  |  |  |

Note: Please put “√” in accord with the options

Quality control supervisor: ____________

Date: _________________

**Table 1. Questionnaire of Demographic Characteristics**

| **No.** | **Questions and options** | **Answers** | |
| --- | --- | --- | --- |
| A1 | **Your nationality?** |  | |
| A2 | **Date of birth?** |  | |
| A3 | **Registered residence?** _________ province/municipality __**_______** city/district/county |  |  |
| A4 | **Household:** ①urban household ②rural household |  | |
| A5 | **Current residence?**_________ province/municipality __**_______** city/district/county |  |  |
| A6 | **How long have you been living in current residence:** ①less than 3 months ② 3 months～6 months ③ 6 months~ 1 year ④ 1 year~ 2 years ⑤ more than two years |  | |
| A7 | **Education level:** ①illiteracy and semiliterate ②primary school ③middle school ④high school/ vocational high school / technical secondary school ⑤ junior college ⑥ college degree and above |  | |
| A8 | **Work place:** ①hotel ②nightclub, bar, dancing hall, teahouse ③massage center, bathing pool, sauna ④barber shop ⑤ street prostitution ⑥other places (please note: __________) |  | |
| A9 | **Marital status:** ①unmarried, no boy friend living together ②unmarried, living with boy friend  ③married ④divorced ⑤widowed ⑥other status (please note: ________) |  | |
| A10 | **Do you have a child (children)?** ①Yes ②No |  | |
| A11 | **The relationships to your family:** ①very well ②well ③general ④bad ⑤very bad |  | |
| A12 | **How do you concern about your own health?** ①very concerned ②concerned ③general  ④not concerned ⑤not concerned at all |  | |

**Table 2. HIV/AIDS knowledge, attitude and behaviors**

| **No.** | **Questions and options** | **Answers** | |
| --- | --- | --- | --- |
| **1. knowledge and attitude** | |  | |
| B1 | **Do you know about HIV/AIDS?** ①today it is the first time that I hear of HIV/AIDS (jump to C1, the investigators explain in detail) ②have heard of, but did not know it very well ③ have heard of, knew some relevant knowledge ④knew it well ⑤knew it very well | Choose①, jump to C1 | |
| B2 | **Do you think the following ways can spread HIV/AIDS?** |  | |
|  | **（1）blood or blood products with HIV:** ①will/maybe ②will not ③don't know |  | |
|  | **（2）sharing needles with HIV-infected persons :** ①will/maybe ②will not ③don't know |  | |
|  | **（3）pregnancy, childbirth or breast feeding:** ①will/maybe ②will not ③don't know |  | |
|  | **（4）having dinner with HIV-infected persons:** ①will/maybe ②will not ③don't know |  | |
|  | **（5）mosquito bites :** ①will/maybe ②will not ③don't know |  | |
|  | **（6）having sex with a person who looks uninfected with HIV:** ①will/maybe ②will not ③don't know |  | |
|  | **（7）using equipments without strict sterilization during a surgery, acupuncture, tooth pulling, beauty therapy, and so on:** ①will/maybe ②will not ③don't know |  | |
|  | **（8）cough, sneeze:** ①will/maybe ②will not ③don't know |  | |
| B3 | **Do you think the following ways can prevent HIV/AIDS?** |  | |
|  | **（1）properly using condoms in every sexual activity:** ①will ②will not ③don't know |  | |
|  | **（2）choosing sexual partners without HIV infection:** ①will ②will not ③don't know |  | |
|  | **（3）using disposable needles:** ①will ②will not ③don't know |  | |
|  | **（4）washing the body before and after sexual activity:** ①will ②will not ③don't know |  | |
|  | **（5）using anti-HIV drugs regularly before and after sexual activity:** ①will ②will not ③don't know |  | |
| B4 | **May unprotected oral sex lead to HIV infection?** ①possible ②impossible ③don't know |  | |
| B5 | **Is there any way to cure HIV/AIDS now?** ①Yes ②No ③don't know |  | |
| B6 | **If someone is infected with HIV, should he/she be isolated?**  ①should be completely ②should be ③hard to say ④should not be ⑤should not be completely |  | |
| B7 | **If someone is infected with HIV, should he/she tell his/her families/spouse?**  ①should tell completely ②should ③hard to say ④should not tell ⑤should not tell completely ⑥according to the individual willingness |  | |
| B8 | **If your friends were infected with HIV, what would you do?**  ①stop contacting with him/her completely ②reduce contacting ③as usual ④care and help him/her ⑤ greatly care and help him/her |  | |
| B9 | **If your spouse/regular sexual partner is infected with HIV, will you keep sex with him/her?**  ① will ②maybe ③hard to say ④maybe not ⑤ will not |  | |
| B10 | **What do you think of the attitude of most people towards HIV-infected persons?**  ①great discrimination ②discrimination ③hard to say ④without discrimination ⑤without discrimination at all |  | |
| B11 | **What**'**s your attitude towards people infected with HIV?**  ①great discrimination ②discrimination ③hard to say ④without discrimination ⑤without discrimination at all |  | |
| B12 | **Do you worry that HIV/AIDS will threat you and your families?**  ①very concerned ②concerned ③general ④not concerned ⑤not concerned at all |  | |
| B13 | **What do you think of the number of people infected with HIV compared to the past?**  ① a great increase ②increase ③nearly same ④decrease ⑤a great decrease |  | |
| B14 | **How do you think of the severity of AIDS?**  ①very serious ②serious ③general ④not serious ⑤not serious at all |  | |
| B15 | **How do you think of the severity of AIDS compared to that of cancer?**  ①much more serious ②more serious ③similar ④less serious ⑤much less serious |  | |
| B16 | **Do you know how many people are infected with HIV among your friends?** (If none, fill in “0”, jump to B19 ) | Fill in“0”, jump to B19 | |
| B17 | **Do you know how they are infected with HIV?**  ①know (please note:________________) ②don't know |  | |
| B18 | **Do you know that their sexual behaviors have any changes after HIV infection?** (multi-choice)  ①Yes, more sex activity than before ②Yes, less than before ③Yes, no longer do sexual services ④Yes, others (please note:________________) ⑤Yes, has no changes ⑥not clear |  | |
| B19 | **Do you know how many people have died from HIV/AIDS among your friends?**  (If none, write“0”) |  | |
| B20 | **Do you know the HIV/AIDS epidemic in the city/district/county where you live?** ①a lot of infections ②a few ③few ④none ⑤not clear |  | |
| B21 | **Do you know the ratio of HIV infection among sex workers in the city/district/county where you live?**  ①very high ②high ③general ④low ⑤very low ⑥none ⑦not clear |  | |
| B22 | **Do you think that the AIDS epidemic will influence the development of the whole society?**  ①absolutely will ②will ③hard to say ④won't ⑤absolutely won't |  | |
| B23 | **Do you think that the AIDS epidemic will influence the life of your family?**  ①absolutely will ②will ③hard to say ④won't ⑤absolutely won't |  | |
| B24 | **Do you think that the AIDS epidemic will influence your life?**  ①absolutely will ②will ③hard to say ④won't ⑤absolutely won't |  | |
| B25 | **Do you agree with the following statements?** |  | |
|  | **（1）If someone is infected with HIV, his/her life is over.**  ①totally agree ②basically agree ③hard to say ④basically disagree ⑤totally disagree |  | |
|  | **（2）If someone is infected with HIV, his/her families will suffer from his/her infection.**  ①totally agree ②basically agree ③hard to say ④basically disagree ⑤totally disagree |  | |
|  | **（3）If someone is infected with HIV, he/she will be too shameful to face others.**  ①totally agree ②basically agree ③hard to say ④basically disagree ⑤totally disagree |  | |
| B26 | **Do you think that the AIDS is still far away from your current life?**  ①very far away ②far away ③hard to say ④not far away ⑤closed to |  | |
| **2. The situation and access to AIDS prevention services** | |  | |
| C1 | **In last 6 months, have you received the following services on AIDS prevention?** |  | |
|  | **（1）condom distribution** ①Yes ②No |  | |
|  | **（2）lubricant distribution** ①Yes ②No |  | |
|  | **（3）materials about knowledge of HIV transmission (booklets, folders and so on)** ①Yes ②No |  | |
|  | **（4）materials about AIDS and venereal diseases (booklets, folders and so on)** ①Yes ②No |  | |
| C2 | **In last 6 months, have you received any free AIDS counseling?**  ①received (jump to C5) ②not received | Choose①,jump to C5 | |
| C3 | **If you never receive any free AIDS counseling, are you willing to have the counseling?**  ①absolutely willing (jump to C5) ②willing (jump to C5) ③hard to say ④unwilling ⑤absolutely willing | Choose①②, jump to C5 | |
| C4 | **If you are unwilling to have free AIDS counseling, why?**  (multi-choice)  ①feel that I don't have any risk of HIV infection ②be afraid that others consider I am in the risk of HIV infection ③don't know where to have the counseling ④don’t want to have counseling at all  ⑤other reasons (please note:______________________________) |  | |
| C5 | **In last 6 months,** **have you ever had an HIV test?** (multi-choice)  ①Yes, I paid for it ②Yes, it was free ③No (jump to C7) | Choose③, jump to C7 | |
| C6 | **Why did you have an HIV test?**  (multi-choice) (jump to C9)  ①I volunteer to do it ②routine testing, such as military recruitment, recruitment, medical examination and blood donation ③have received medical services, such as before a surgery operation ④ CDC-organized campaigns ⑤others (please note: ) | Jump to C9 | |
| C7 | **Would you like to have a free HIV test if you haven't had one before?** (If you choose①/②, please jump to C9)  ① absolutely willing ②willing ③ hard to say ④unwilling ⑤ absolutely unwilling | Choose①②, jump to C9 | |
| C8 | **If not, why?**  (multi-choice)  ①feel no risk of HIV infection ②be afraid that others know I have risk to infect HIV ③don't know where to have the test ④ fell embarrassed to have an HIV test ⑤ be afraid to face HIV infection or afraid of HIV test ⑥others (please note: ) |  | |
| C9 | **What is your primary way to obtain information about HIV/AIDS?** (multi-choice)  ①network ②TV ③broadcast ④doctors ⑤relatives ⑥friends ⑦newspapers and magazines  ⑧books ⑨school education ⑩publicity materials ⑾theatrical performances  ⑿advertising column ⒀consulting service ⒁drug treatment center, methadone clinic, and so on  ⒂peer education ⒃unclear ⒄others (please note: ) ⒅never obtain |  | |
| **C10** | **Are you willing to learn about the knowledge of AIDS prevention?**  (If you choose④/⑤, please jump to D1)  ① absolutely willing ②willing ③hard to say ④unwilling (jump to D1) ⑤ absolutely unwilling (jump to D1) | Choose④⑤, jump to D1 | |
| C11 | **How would you like to learn more information on AIDS?** (multi-choice)  ①network ②TV ③broadcast ④doctors ⑤relatives ⑥friends ⑦newspapers and magazines  ⑧books ⑨school education ⑩publicity material ⑾theatrical performances  ⑿advertising column ⒀consulting service ⒁drug treatment center, methadone clinic, and so on  ⒂peer education ⒃unclear ⒄others (please note: ) ⒅unwilling to obtain |  | |
| **3. Behavioral characteristics** | |  | |
| D1 | **How old were you when you first had sex?** years old |  | |
| D2 | **How long have you done this occupation?** years months |  |  |
| D3 | **How long have you worked here?** years months |  |  |
| D4 | **Your current monthly income:**  ①~1000 yuan and below ②1001 ~ 3,000 yuan ③3001 ~ 5,000 yuan ④5001 ~ 7,000 yuan  ⑤7001 ~ 9,000 yuan ⑥more than 9,000 yuan |  | |
| D5 | **How often did you change a workplace?**  ①less than three months ②three months~ ③six months~ ④one year~ ⑤never |  | |
| D6 | **How many clients per week?** |  | |
| D7 | **The maximum number of clients did you have one day?** |  | |
| **D8** | **How often did you use condoms when having sex with clients in the past six months?** (vaginal intercourse, anal intercourse)  ①never ②occasionally (less than 20%) ③small part of the time (20% ~ 40%)  ④half of the cases (40% ~ 60%) ⑤most of the time (60% ~ 80%) ⑥frequently (more than 80%)  ⑦every time (100%) (jump to D10) | Choose⑦, please jump to D10 | |
| D9 | **The reasons that you didn’t use condoms when having sex with clients?** (multi-choice)  ①(at that time) never thought to use a condom ②no condoms available around at that time ③too expensive ④using condoms is not comfortable ⑤clients refused to use condoms ⑥clients paid more without using condoms ⑦feared that he thinks I don't trust him ⑧was embarrassed or afraid to ask him to use ⑨the partner didn’t seem to have sexually transmitted diseases ⑩had taken other contraceptive measures ⑾had taken other security measures (please note: ) ⑿others (please note: ) |  | |
| D10 | **How many times have you uses condoms when having sex with clients in the last 3 sexual intercourses?**  ①never used ②once ③twice ④three times |  | |
| D11 | **How often did you use condoms when having oral sex with clients in the past six months?**  ①never ②occasionally (less than 20%) ③small part of the time (20% ~ 40%)  ④half of the cases (40% ~ 60%) ⑤most of the time (60% ~ 80%) ⑥frequently (more than 80%)  ⑦every time (100%) (jump to D13) ⑧ never had a oral sex (jump to D13) | Choose⑦⑧, jump to D13 | |
| D12 | **The reasons that you didn’t use condoms when having oral sex with clients?**  (multi-choice)  ①(at that time) never thought to use a condom ②no condoms available around at that time ③too expensive ④using condoms is not comfortable ⑤clients refused to use condoms ⑥clients paid more without using condoms ⑦feared that he thought that I didn’t trust him ⑧was embarrassed or afraid to ask him to use ⑨the partner didn’t seem to have sexually transmitted diseases ⑩oral sex doesn’t spread sexually transmitted diseases and HIV ⑾had take other security measures (please note: )  ⑿others (please note: ) |  | |
| D13 | **What’s the attitude of gatekeeper towards using condoms?**  ①request to use ②doesn’t request, but suggest use  ③doesn’t care, it is our own business ④request not to use |  | |
| D14 | **Did the gatekeeper force you to use condoms when you had sex with clients?**  ①Yes ②No ③hard to say |  | |
| D15 | **How often did you have sex with your spouse/fixed sex partner in the past six months?**  ①once or more a week ②once every two weeks ③once every month ④less than once a month  ⑤did not happen (jump to D21) ⑥unmarried or without a fixed sex partner (jump to D21) | Choose⑤⑥, jump to D21 | |
| D16 | **How often did you use condoms when having sex with your spouse/fixed sex partner in the past six months?** (vaginal intercourse, anal intercourse) (If you choose⑦, please jump to D18)  ①never ②occasionally (less than 20%) ③small part of the time (20% ~ 40%)  ④half of the cases (40% ~ 60%) ⑤most of the time (60% ~ 80%) ⑥frequently (more than 80%)  ⑦every time (100%) (jump to D18) | Choose⑦, jump to D18 | |
| D17 | **The reasons that you didn’t use a condom when having sex with your spouse/fixed sex partner?** (vaginal intercourse, anal intercourse) (multi-choice)  ①(at that time) never thought to use a condom ②no condoms available around at that time ③too expensive ④using condoms is not comfortable ⑤ the spouse/partner refused to use condoms ⑥ was embarrassed or afraid to ask him to use ⑦ was afraid that the spouse/partner suspected that I had sexually transmitted diseases ⑧had a plan to have a child ⑨ had taken other contraceptive measures ⑩ had taken other security measures (please note: ) ⑾others (please note: ) |  | |
| D18 | **Did you use a condom the last time when having sex with your spouse/fixed sex partner?**  (vaginal intercourse, anal intercourse) ①Yes ②No |  | |
| D19 | **How often did you use condoms when having oral sex with your spouse/fixed sex partner in the past six months?**  ①never ②occasionally (less than 20%) ③small part of the time (20% ~ 40%)  ④half of the cases (40% ~ 60%) ⑤most of the time (60% ~ 80%) ⑥frequently (more than 80%)  ⑦every time (100%) (jump to D21) ⑧not occurred (jump to D21) | Choose⑦⑧, jump to D21 | |
| D20 | **The reasons that you didn’t use a condom when having oral sex with your spouse/fixed sex partner?**  ①(at that time) never thought to use a condom ②no condoms available around at that time ③too expensive ④using condoms is not comfortable ⑤ the spouse/partner refused to use condoms ⑥ was embarrassed or afraid to ask him to use ⑦ was afraid that the spouse/partner suspected that I had sexually transmitted diseases ⑧believed that the spouse/partner had no infection ⑨ oral sex does not spread sexually transmitted diseases and AIDS ⑩had take other security measures (please note: ) ⑾others (please note: ) |  | |
| D21 | **How many temporary sex partners did you have in the past six months?**  (If none, jump to D27) | Fill in “0”, jump to D27 | |
| D22 | **How often did you use condoms when having sex with temporary sex partners in the past six months?** (vaginal intercourse, anal intercourse)  ①never ②occasionally (less than 20%) ③small part of the time (20% ~ 40%)  ④half of the cases (40% ~ 60%) ⑤most of the time (60% ~ 80%) ⑥frequently (more than 80%)  ⑦every time (100%) (jump to D24) | Choose⑦, jump to D24 | |
| D23 | **The reasons you didn’t use a condom when having sex with temporary sex partner?**  (vaginal intercourse, anal intercourse) (multi-choice)  ①(at that time) never thought to use a condom ②no condoms available around at that time ③too expensive ④using condoms is not comfortable ⑤the partner refused to use condoms ⑥ feared that he thought that I didn't trust him ⑦was embarrassed or afraid to ask him to use ⑧believed that the partner had no infection ⑨was afraid that the partner suspected that I had sexually transmitted diseases ⑩had take other contraceptive measures ⑾had taken other security measures (please note: ) ⑿others (please note: ) |  | |
| D24 | **Did you use a condom the last time when having sex with temporary sex partner?**  (vaginal intercourse, anal intercourse) ① Yes ②No |  | |
| D25 | **How often did you use condoms when having oral sex with temporary sex partner in the past six months?**  ①never ②occasionally (less than 20%) ③small part of the time (20% ~ 40%)  ④half of the cases (40% ~ 60%) ⑤most of the time (60% ~ 80%) ⑥frequently (more than 80%)  ⑦every time (100%) (jump to D27) ⑧not occurred (jump to D27) | Choose⑦⑧, jump to D27 | |
| D26 | **The reasons that you didn’t use a condom when having oral sex with temporary sex partner?** (multi-choice)  ①(at that time) never thought to use a condom ②no condoms available around at that time ③too expensive ④using condoms is not comfortable ⑤the partner refused to use condoms ⑥feared that he thought I didn't trust him ⑦was embarrassed or afraid to ask him to use ⑧believed that the partner had no infection ⑨ oral sex doesn’t spread sexually transmitted diseases/AIDS ⑩had take other security measures (please note: ) ⑾others (please note: ) |  | |
| D27 | **Did you use condoms throughout when having sex with others?**  ①never ②occasionally (less than 20%) ③small part of the time (20% ~ 40%)  ④half of the cases (40% ~ 60%) ⑤most of the time (60% ~ 80%) ⑥frequently (more than 80%)  ⑦every time (100%) (jump to D29) | Choose⑦, jump to D29 | |
| D28 | **Why didn’t you use condoms throughout when having sex with others?** (multi-choice)  ①condom is only used for contraception and only used before ejaculation  ②sometimes condom sticks in the vagina  ③only semen can spread sexually transmitted diseases and HIV, just use condoms before ejaculation  ④believe that the spouse has no infection, so did not use condoms throughout when having sex with spouse  ⑤believe that the temporary sex partners have no infection, so did not use condoms throughout when having sex with temporary sex partners  ⑥believe that regular clients have no infection, so did not use condoms throughout when having sex with regular clients  ⑦the clients do not seem to have a disease  ⑧the partners are unwilling to use condoms  ⑨others (please note: ) |  | |
| D29 | **Is there anyone who had sex with you is HIV-positive?**  ①Yes ②No ③not sure |  | |
| D30 | **Have you ever had the following symptoms of venereal diseases?**  (micturition pain or burning sensation, abnormal male urethral secretions or abnormal female vaginal secretions, genital skin damage, ulcers, dyspareunia, severe or persistent pelvic pain ) ①Yes ②No (jump to D32) | Choose②, jump to D32 | |
| D31 | **How do you solve the symptoms of venereal disease usually?**  ①to venereal disease specialist clinics ②to hospitals above the county level  ③to maternal and children’s hospitals ④to private clinics ⑤to township hospitals or community health center ⑥to village health or community health service stations ⑦buy some drugs  ⑧no treatment ⑨others (please note: ) |  | |
| D32 | **Have you ever been diagnosed with STD?**  ①Yes ②No (skip to D35) |  | |
| D33 | **What kind of diseases did you have?** (multi-choice)  ①syphilis ②genital herpes ③gonorrhea ④verruca acuminata ⑤non-specific urethritis  ⑥others (please note: ) ⑦not clear |  | |
| D34 | **The last time you got a STD?**  ①within one year ②one year ago ③three years ago ④five years ago |  | |
| **D35** | **How often have you drunk alcohol in the past six months?**  ( If you choose⑤, please skip to D37)  ①almost every day ②at least three times a week ③at least once a week  ④less than once a week ⑤never drink (jump to D37) | Choose⑤, jump to D37 | |
| D36 | **Did you use alcohol before you had sex with partners?**  ①never use ②occasionally (less than 20%) ③small part of the time (20%~ 40%)  ④half of the cases (40% ~ 60%) ⑤most of the time (60% ~ 80%) ⑥frequently (more than 80%)  ⑦every time (100%) |  | |
| D37 | **Have you ever used the following substances in the past six months?**  ①No (skip to D42) ②ecstasy ③methamphetamine ④ketamine ⑤opium ⑥marijuana ⑦heroin  ⑧MaGu ⑨pethidine ⑩morphine ⑾others (please note: ) | Choose①, jump to D42 | |
| D38 | **Have you ever used the above substances before having sex with partner in the past six months?**  ①Yes ②No (jump to D40) | Choose②, jump to D40 | |
| D39 | **How often have you used the above substances before having sex with partner in the past six months?**  ①never use ②occasionally (less than 20%) ③small part of the time (20%~ 40%)  ④half of the cases (40% ~ 60%) ⑤most of the time (60% ~ 80%) ⑥frequently (more than 80%)  ⑦every time (100%) |  | |
| D40 | **Have you ever injected the above substances in the past six months?**  ①Yes ②No (jump to D42) | Choose②, jump to D42 | |
| D41 | **Do you know any person who has shared needles with you is now found to be HIV positive?**  ①Yes ②No ③not clear ④not had shared needles with others |  | |
| D42 | **How do you think of your risk of HIV infection?**  ①very high ②high ③ in general ④low ⑤very low |  | |
| D43 | **What do you think that your HIV risk mainly comes from:_______** (multi-choice, and sort)  ①[unfamiliar](app:lj:不熟悉的?ljtype=blng&ljblngcont=0&ljtran=unfamiliar) clients ②familiar clients ③temporary sexual partners ④spouse/fixed sexual partner  ⑤others (please note: _______________) |  | |

**Table 3. Knowledge, attitude and use intention of HIV prevention methods**

| **NO.** | **Question and options** | | | | **Answers** | | |
| --- | --- | --- | --- | --- | --- | --- | --- |
| **E1** | **Do you think that it is difficult to prevent AIDS currentiy?** ①very hard ②hard ③in general ④not hard ⑤not hard at all | | | |  | | |
| **E2** | **Do you think that you have the ability to prevent AIDS?**  ①absolutely have the ability ②have the ability ③hard to say ④lack of ability ⑤have no ability at all | | | |  | | |
| **E3** | **Do you agree with the following statements?** | | | |  | | |
|  | 1. **Very few male persons are likely to use condoms**   ①absolutely agree ②agree ③hard to say ④disagree ⑤absolutely disagree | | | |  | | |
|  | 1. **You will suggest use condom if your partners don’t reject to use it**   ①absolutely agree ②agree ③hard to say ④disagree ⑤absolutely disagree | | | |  | | |
|  | 1. **The clients will consider you have a sexual disease if you insist on using condoms**   ①absolutely agree ②agree ③hard to say ④disagree ⑤absolutely disagree | | | |  | | |
|  | 1. **You can persuade the partner to use condoms even he is unwilling to**   ①absolutely agree ②agree ③hard to say ④disagree ⑤absolutely disagree | | | |  | | |
|  | 1. **You will refuse to have sex with the clients who reject to use condoms**   ①absolutely agree ②agree ③hard to say ④disagree ⑤absolutely disagree | | | |  | | |
|  | 1. **You know how to use condoms correctly**   ①absolutely agree ②agree ③hard to say ④disagree ⑤absolutely disagree | | | |  | | |
|  | 1. **The clients will be angry if you insist on using condoms**   ①absolutely agree ②agree ③hard to say ④disagree ⑤absolutely disagree | | | |  | | |
|  | 1. **The clients will no longer have sex with you if you insist on using condoms**   ①absolutely agree ②agree ③hard to say ④disagree ⑤absolutely disagree | | | |  | | |
|  | 1. **The clients will pay more money if you don’t use condoms**   ①absolutely agree ②agree ③hard to say ④disagree ⑤absolutely disagree | | | |  | | |
|  | 1. **No using condom can save time and money**   ①absolutely agree ②agree ③hard to say ④disagree ⑤absolutely disagree | | | |  | | |
|  | 1. **Using condom can prevent you infecting with sexually transmitted diseases and HIV**   ①absolutely agree ②agree ③hard to say ④disagree ⑤absolutely disagree | | | |  | | |
| **E4** | **Have you ever heard of the following HIV prevention methods? What do you think of the possibility of their prevention effects?**  (investigator explains the following measures) | | | |  | | |
|  | 1. **Vagina/Rectum microbicide** ①have heard of ②never heard of | | | |  | | |
|  | **the possibility of its prevention effects:** ①very good ②good ③common ④poor ⑤very poor | | | |  | | |
|  | **（2）Pre-exposure prophylaxis** ①have heard of ②never heard of | | | |  | | |
|  | **the possibility of its prevention effects:** ①very good ②good ③common ④poor ⑤very poor | | | |  | | |
|  | **（3）Post-exposure prophylaxis** ①have heard of ②never heard of | | | |  | | |
|  | **the possibility of its prevention effects:** ①very good ②good ③common ④poor ⑤very poor | | | |  | | |
| **E5** | **Have you ever used drugs to prevent STD infection?** ①Yes ②No (jump to E10) | | | | Choose②, jump to E10 | | |
| **E6** | **What drugs did you use?** | | | |  | | |
| **E7** | **The usage of drugs:** ①oral use ②external use ③injection | | | |  | | |
| **E8** | **Who suggests you use the drugs/who offers you the drugs?** (multi-choice)  **①**doctors ②family members ③sexual partners ④friends ⑤used by self ⑥ others (please note: _______) | | | |  | | |
| **E9** | **The time of using drugs: (multi-choice)**  ①before sexual behavior ②after sexual behavior ③(multiple) regularly use during the period of sexual behavior | | | |  | | |
| **E10** | **Have you ever used drugs to prevent HIV?** ①Yes ②No (jump to E15) | | | | Choose②, jump to E15 | | |
| **E11** | **What drugs did you use?** | | | |  | | |
| **E12** | **The usage of drugs:** ①oral use ②external use ③injection | | | |  | | |
| **E13** | **Who suggests you to use the drugs/who offers you the drugs?** (multi-choice)  **①**doctors ②family members ③sexual partners ④friends ⑤used by self ⑥ others (please note: _______) | | | |  | | |
| **E14** | **The time of using drugs: (multi-choice)**  ①before sexual behavior ②after sexual behavior ③(multiple) regularly use during the period of sexual behavior | | | |  | | |
| **E15** | **Do you know anyone who has used drugs to prevent HIV around?** ①Yes ②No (jump to E19) | | | | Choose②, jump to E19 | | |
| **E16** | **What drugs did he/she use?** | | | |  | | |
| **E17** | **The usage of drugs:** ①oral use ②external use ③injection | | | |  | | |
| **E18** | **The time of using drug: (multi-choice)**  ①before sexual behavior ②after sexual behavior  ③(multiple) regularly use during the period of sexual behavior | | | |  | | |
| **E19** | **How do you think of the possibility of HIV infection if you don’t take any prevention measure when you have sex with your spouse/fixed sexual partners?**  ①very high ②high ③common ④low ⑤very low ⑥have no spouse/fixed sexual partner | | | |  | | |
| **E20** | **How do you think of the possibility of HIV infection if you don’t take any prevention measure when you have sex with your clients?**  ①very high ②high ③common ④low ⑤very low | | | |  | | |
| **E21** | **How do you think of the possibility of HIV infection if you don’t take any prevention measurs when you have sex with your temporary sexual partners?**  ①very high ②high ③common ④low ⑤very low ⑥have no temporary sexual partner | | | |  | | |
| **E22** | **Do you think that PrEP should be promoted among sex workers if the safety and effectiveness of PrEP have been proved?**  ①absolutely should be ②should be ③hard to say ④shouldn’t be ⑤absolutely shouldn’t be | | | |  | | |
| **E23** | **What are your attitudes about the followings statements?** | | | |  | | |
|  | **（1）If PrEP were safe and effective, how likely would you be willing to use it?**  ①absolutely willing (jump to E25) ②possibly willing (jump to E25) ③unknown ④possibly unwilling ⑤absolutely unwilling | | | | Choose①②, jump to E25 | | |
|  | **（2）If PrEP were safe, effective and free, how likely would you be willing to use it?**  ①absolutely willing (jump to E25) ②possibly willing (jump to E25) ③unknown ④possibly unwilling ⑤absolutely unwilling | | | | Choose①②, jump to E25 | | |
|  | **（3）If PrEP were safe, effective, free and being used by few people around you, how likely would you be willing to use it?**  ①absolutely willing (jump to E25) ②possibly willing (jump to E25) ③unknown ④possibly unwilling ⑤absolutely unwilling | | | | Choose①②, jump to E25 | | |
|  | **（4）If PrEP were safe, effective, free and being used by many people around you, how likely would you be willing to use it?**  ①absolutely willing (jump to E25) ②possibly willing (jump to E25) ③unknown ④possibly unwilling ⑤absolutely unwilling | | | | Choose①②, jump to E25 | | |
| **E24** | **The reasons that you are unwilling to accept pre-exposure prophylaxis:** (multi-choice)  ①no risk of HIV infection, not necessary ②worry about the side effects of drugs ③worry about drugs having no effects ④worry about discrimination by others ⑤worry about objections of clients ⑥worry about objection of families ⑦worry about the objections of gatekeepers ⑧Others (please note: _____________) | | | |  | | |
| **E25** | **If PrEP were effective and safe，are you willing to suggest your friend accept it?**  ①absolutely willing ②possibly willing ③unknown ④possibly unwilling ⑤absolutely unwilling | | | |  | | |
| **If the respondent is unwilling to take pre-exposure prophylaxis (E23(4) choose④or⑤), investigator should end this part of the questionnaire, and jump to psychological personality scale.** | | | | |  | | |
| E26 | **What are your concerns about PrEP?** (multi-choice)  ①effect (effective ) ②safety (side effects) ③cost ④convenience of acquiring drugs ⑤convenience of taking drugs ⑥attitudes of the people around ⑦support of sexual partners ⑧support of families ⑨the people around use drugs or not ⑩others (please note: ______________) I don’t care at all | | | |  | | |
| E27 | **Do you worry about being found by others when you use PrEP? Who do you worry about?** (Multi-choice)  ①No, I don’t worry ②the clients ③the spouse/fixed sexual partners ④temporary sexual partners ⑤family members ⑥friends ⑦gatekeepers ⑧others (please note: ______________) | | | |  | | |
| E28 | **The attitudes of the following persons on your PrEP using?**  ①support ②not clear ③objection ④ not these persons | spouse/fixed sexual partners | temporary sexual partners | clients | family members | friends | gatekeepers |
|  |  |  |  |  |  |  |  |
|  |  | (If the answer is④, jump to the next question) | | | | | |
| E29 | **Will the following persons’ attitudes influence your choice?**  ①absolutely will ②possibly will ③not clear ④possibly won’t ⑤absolutely won’t |  |  |  |  |  |  |
| E30 | **What are your clients’ attitudes if he knows about your PrEP using?**  (multi-choice)  ①angry ②no longer have sex with you ③feel more secure ④consider that you are infected ⑤ask you not to use PrEP ⑥others (please note: _______ ) | | | |  | | |
| E31 | **Do you agree with the following statements?** | | | |  | | |
|  | 1. **You will be discriminated by others if they know about your using of PrEP?**   ①absolutely agree ②agree ③hard to say ④disagree ⑤absolutely disagree | | | |  | | |
|  | 1. **I convince that I can insist on PrEP no matter what others’ attitudes are.**   ①absolutely agree ②agree ③hard to say ④disagree ⑤absolutely disagree | | | |  | | |
| E32 | **How much would you like to pay for PrEP if it is not free?**  ①none ②<100 yuan ③100~ yuan ④200~ yuan ⑤400~ yuan ⑥600~ yuan ⑦800~ yuan ⑧>1000 yuan | | | |  | | |
| E33 | **Will you insist on PrEP if you need to take medicine every day?**  ①absolutely will (jump to E35) ②possibly will (jump to E35) ③not clear ④possibly won’t  ⑤absolutely won’t | | | | Choose①②, jump to E35 | | |
| E34 | **How often can you insist on PrEP using if you don’t want to take medicine every day?**  ①once every two days ②once every three days ③once a week ④half a month ⑤once a month  ⑥others (please note: _______ ) | | | |  | | |
| E35 | **If there are two medicines, one is cheap and has a short medication period, while the other is expensive and has a long medication period, which one do you prefer to?**  ①first one ②second one ③have no idea | | | |  | | |
| E36 | **How would you like to get more information of PrEP?**  (multi-choice)  ①internet ②television ③broadcast ④doctor ⑤relatives ⑥friends ⑦news papers and magazines ⑧books ⑨school education ⑩publicity material ⑾live shows ⑿advertisements ⒀counseling service ⒁detoxification center/methadone clinic and so on ⒂peer education group ⒃not clear ⒄other (please note: _______ ) ⒅don’t want to know | | | |  | | |
| E37 | **Where would you like to get the PrEP medicines?**  (multi-choice)  ①village health clinics/health service station ②health clinics in towns and townships/health service center ③hospitals at or above the county levels ④centers for disease control and prevention (CDC) ⑤AIDS counseling service organizations ⑥sexually transmitted disease outpatients ⑦detoxification center/methadone clinic ⑧private clinic ⑨drugstores ⑩adult health care shop ⑾others (please note: _______ ) | | | |  | | |
| E38 | **Why do you want to get the PrEP medicine at this place(s)?**  (multi-choice)  ①It has a strong protection of privacy. ②convenient ③high level of medical care ④others  (please note: _______ ) | | | |  | | |
| E39 | **What do you think of the frequency of condom use when you use PrEP simultaneously?**  ①absolutely rise ②possibly rise ③as usual ④possibly decrease ⑤absolutely decrease | | | |  | | |
| E40 | **What do you think of the number of your sexual partners if you use PrEP?**  ①absolutely rise ②possibly rise ③as usual ④possibly decrease ⑤absolutely decrease | | | |  | | |
| E41 | **Would you like to participate in a PrEP clinical trial about its safety and efficacy?**  ①absolutely willing (only answer the E42) ②willing (only answer the E42) ③hard to say (answer both E42 and E43) ④unwilling (jump to E43) ⑤absolutely willing (jump to E43) | | | | Choose④⑤, jump to E43 | | |
| E42 | **The reasons that you are willing to** **participate in a PrEP clinical trial :** (multi-choice)  ①worry about the HIV risk ②hope to promote PrEP prevention method ③free to join the trial, and free to take prevention drugs ④others (please note: _______ ) | | | |  | | |
| E43 | **The reasons that you are not willing to participate in a PrEP clinical trial:** (multi-choice)  ①no risk of HIV infection ②worry about the side effects of drugs ③worry about drugs having no effects ④worry about discrimination by others ⑤worry about objections of sexual partners ⑥worry about objection of families ⑦others (please note: _______ ) | | | |  | | |
