# Supplementary material for: HIV Pre-Exposure Prophylaxis Interest among Female Sex Workers in Guangxi, China
Source: PLoS One. 2014 Jan 22;9(1):e86200. doi: 10.1371/journal.pone.0086200 (PMC3899205; doi:10.1371/journal.pone.0086200)
Supplement: Document S4 — (DOC) [file pone.0086200.s004.doc]

**定量调查问卷**

导语：

您好！我们是国家重大专项——艾滋病暴露前用药可行性研究课题的调查员，这次调查主要是了解您对健康知识、态度、行为的认识，可能会涉及到您某些隐私，我们会对您回答的内容严格保密，只做人群科研分析，不做个人分析，也不会向其他任何个人或单位透露您的信息，希望您能够如实回答下面的问题。本次调查可能会耽搁您一些时间，请您谅解！非常感谢您的支持！

**问卷基础信息**

问卷编号：**□□□□□□□□□□**（行政区划国标码+调查对象类别+调查对象编号）

调查地点：___________省（直辖市）___________市/区/县

调查场所：____________

调查对象知情同意：_______________（是/否）

调查员：____________

调查日期：______年______月______日

表 调查问卷质量监督表

| 项目 | 完成情况 | | 备注 |
| --- | --- | --- | --- |
|  | 是 | 否 |  |
| 问卷填写规范、清晰 |  |  |  |
| 每个问题都得到了回答，无漏项 |  |  |  |
| 无逻辑错误 |  |  |  |

注：请在符合的选项下打“√”

调查问卷质量监督员：____________

质量监督日期：______年______月______日

表1. 一般情况调查表

| **序号** | **问题及选项** | **回答** | |
| --- | --- | --- | --- |
| A1 | **您的民族？** |  | |
| A2 | **您的出生年份？** |  | |
| A3 | **您的户籍所在地？** _________省/直辖市__**_______**市/区/县 |  |  |
| A4 | **您的户口是：** ①城镇户口 ②农村户口 |  | |
| A5 | **目前居住地？**_________省/直辖市__**_______**市/区/县 |  |  |
| A6 | **在目前居住地居住时间：** ①＜3月 ②3～月 ③6～月 ④1～年 ⑤2年及以上 |  | |
| A7 | **文化程度：**①文盲与半文盲 ②小学 ③初中 ④高中/职高/中专 ⑤大专 ⑥大学本科及以上 |  | |
| A8 | **工作地点：**①宾馆 ②酒吧、歌舞厅、茶室、会所 ③发廊 ④浴池、桑拿、足疗、按摩 ⑤街头(站桩) ⑥路边店 ⑦其它（请注明_____________） |  | |
| A9 | **婚姻状况：**  ①未婚，无同居男友 ②未婚，有同居男友 ③已婚 ④离异 ⑤丧偶 ⑥其它（请注明________） |  | |
| A10 | **现在是否有小孩：** ①有 ②无 |  | |
| A11 | **自我感觉目前与家人的关系：** ①非常好 ②好 ③一般 ④差 ⑤非常差 |  | |
| A12 | **您认为自己对自我健康的关心程度怎样？** ①非常关心 ②关心 ③一般 ④不关心 ⑤非常不关心 |  | |

**表2. 艾滋病知识、态度、行为**

| **序号** | **问题及选项** | **回答** | |
| --- | --- | --- | --- |
| **1、知识、态度** | |  | |
| B1 | **您知道艾滋病吗？** ①今天是头一回听说（跳至C1，调查员简单解释艾滋病） ②听说过，但具体是怎么回事不清楚 ③听说过，也了解一些相关知识 ④知道，我比较了解这个病 ⑤知道，我非常了解这个病 | （选①，跳至C1） | |
| B2 | **您认为下列哪些方式可以传播艾滋病？** |  | |
|  | **（1）输入带有艾滋病病毒的血液或血液制品** ①会/可能 ②不会 ③不知道 |  | |
|  | **（2）与艾滋病病毒感染者或病人共用针具** ①会/可能 ②不会 ③不知道 |  | |
|  | **（3）感染了艾滋病病毒的孕妇怀孕、分娩、哺乳** ①会/可能 ②不会 ③不知道 |  | |
|  | **（4）与艾滋病病毒感染者或病人一起吃饭** ①会/可能 ②不会 ③不知道 |  | |
|  | **（5）蚊虫叮咬** ①会/可能 ②不会 ③不知道 |  | |
|  | **（6）与看起来干净、健康的人发生性关系** ①会/可能 ②不会 ③不知道 |  | |
|  | **（7）使用未严格消毒的手术、针灸、拔牙、美容等器械** ①会/可能 ②不会 ③不知道 |  | |
|  | **（8）咳嗽、打喷嚏** ①会/可能 ②不会 ③不知道 |  | |
| B3 | **您认为下列哪些方式可以预防艾滋病？** |  | |
|  | **（1）每次正确使用质量合格的安全套** ①可以 ②不可以 ③不知道 |  | |
|  | **（2）保持一个未感染艾滋病病毒的性伴** ①可以 ②不可以 ③不知道 |  | |
|  | **（3）使用一次性针具** ①可以 ②不可以 ③不知道 |  | |
|  | **（4）性行为前后清洗下身** ①可以 ②不可以 ③不知道 |  | |
|  | **（5）性行为前后规律服用抗艾滋病病毒的药物** ①可以 ②不可以 ③不知道 |  | |
| B4 | **无保护的口交是否可能感染艾滋病？** ①可能 ②不可能 ③不知道 |  | |
| B5 | **得了艾滋病，现在有没有办法治好（愈）？** ①有 ②没有 ③不知道 |  | |
| B6 | **一个人感染了艾滋病病毒，是否应该隔离她/他？**  ①完全应该 ②应该 ③不好说 ④不应该 ⑤完全不应该 |  | |
| B7 | **一个人感染了艾滋病病毒，是否应该告诉他的家人/配偶？**  ①完全应该 ②应该 ③不好说 ④不应该 ⑤完全不应该 ⑥尊重个人意愿 |  | |
| B8 | **如果您朋友感染了艾滋病病毒，您会怎样？**  ①完全断绝往来 ②减少来往 ③和以前一样 ④关心帮助他 ⑤非常关心帮助他 |  | |
| B9 | **如果您的配偶/固定性伴感染了艾滋病病毒，您是否和她/他继续保持性关系？**  ①肯定会 ②很可能会 ③不好说 ④很可能不会 ⑤肯定不会 |  | |
| B10 | **您认为大多数人对艾滋病感染者/病人的态度怎样？**  ①非常歧视 ②歧视 ③不好说 ④不歧视 ⑤完全不歧视 |  | |
| B11 | **您对艾滋病感染者/病人的态度怎样？** ①非常歧视 ②歧视 ③不好说 ④不歧视 ⑤完全不歧视 |  | |
| B12 | **您是否担心艾滋病会对您和您的家人造成威胁？**  ①非常担心 ②担心 ③不好说 ④不担心 ⑤完全不担心 |  | |
| B13 | **您认为现在的艾滋病感染者数量与过去相比怎样？**  ①有很大增加 ②有所增加 ③差不多 ④有所减少 ⑤有很大减少 |  | |
| B14 | **您认为艾滋病的严重程度如何？** ①非常严重 ②严重 ③一般 ④不严重 ⑤完全不严重 |  | |
| B15 | **与癌症相比，您认为艾滋病的严重程度如何？**  ①比癌症严重得多 ②比癌症严重 ③和癌症差不多 ④没有癌症严重 ⑤远没有癌症严重 |  | |
| B16 | **您知道或认识您周围朋友中有多少人感染了艾滋病病毒？**（若无，填“0”，跳至B19） | （填“0”，跳至B19） | |
| B17 | **您知道她们是如何感染的吗？**  ①知道（请注明________________） ②不知道 |  | |
| B18 | **您知道她们感染以后性行为有什么改变吗？**（可多选）  ①有，比以前更多的通过性服务赚钱 ②有，比以前的性服务减少了 ③有，不再做性服务了 ④有，其它（请注明________________） ⑤没有什么改变 ⑥不清楚 |  | |
| B19 | **您知道或认识您周围朋友中有多少人因艾滋病感染而死亡？**（若无，填“0”） |  | |
| B20 | **您认为您所生活的市/区/县是否有艾滋病感染者/病人。**①有较多 ②有少数 ③没有 ④不清楚 |  | |
| B21 | **您认为您所生活的市/区/县的性工作者中是否有艾滋病感染者/病人，性工作者艾滋病感染的比率怎样？**①很高 ②高 ③一般 ④低 ⑤非常低 ⑥没有感染者/病人 ⑦不清楚 |  | |
| B22 | **您认为艾滋病流行会影响整个社会的发展吗？** ①肯定会 ②会 ③不好说 ④不会 ⑤肯定不会 |  | |
| B23 | **您认为艾滋病流行会影响您的家庭生活吗？** ①肯定会 ②会 ③不好说 ④不会 ⑤肯定不会 |  | |
| B24 | **您认为艾滋病流行会对您个人造成影响吗？** ①肯定会 ②会 ③不好说 ④不会 ⑤肯定不会 |  | |
| B25 | **您是否同意下列说法** |  | |
|  | **（1）如果一个人得了艾滋病，那他/她这一辈子就完了**  ①完全同意 ②基本同意 ③不好说 ④基本不同意 ⑤完全不同意 |  | |
|  | **（2）如果一个人得了艾滋病，全家人多会跟着他/她倒霉**  ①完全同意 ②基本同意 ③不好说 ④基本不同意 ⑤完全不同意 |  | |
|  | **（3）如果一个人得了艾滋病，他/她就没脸面对其他人**  ①完全同意 ②基本同意 ③不好说 ④基本不同意 ⑤完全不同意 |  | |
| B26 | **您认为艾滋病离你现在的生活遥远吗？** ①非常遥远 ②遥远 ③不好说 ④不遥远 ⑤非常不遥远 |  | |
| **2、获得预防艾滋病服务的情况** | |  | |
| C1 | **最近半年，您是否接受过有关预防艾滋病的下列服务** |  | |
|  | **（1）安全套发放** ①是 ②否 |  | |
|  | **（2）润滑剂发放** ①是 ②否 |  | |
|  | **（3）伙伴之间的相关知识传播** ①是 ②否 |  | |
|  | **（4）艾滋病性病宣传材料（小册子、折页等）** ①是 ②否 |  | |
| C2 | **最近一年，您接受过艾滋病免费咨询吗？** ①接受过（跳至C5） ②没接受过 | （选①，跳至C5） | |
| C3 | **如果您没做过，您愿意做吗？**  ①非常愿意（跳至C5） ②愿意（跳至C5） ③不好说 ④不愿意 ⑤非常不愿意 | （选①、②，跳至C5） | |
| C4 | **如果您不愿意，是为什么？**（可多选）  ①觉得自己没有感染艾滋病的风险 ②害怕别人知道自己去做咨询 ③不知道在哪里能做该咨询 ④不好意思去做艾滋病咨询 ⑤其它（______________________________） |  | |
| C5 | **最近一年，您做过艾滋病病毒检测吗？** （可多选）  ①做过，收费的 ②做过，免费的 ③没做过（跳至C7） | （选③，跳至C7） | |
| C6 | **您做检测时，是在以下哪种情形中发生的？**（可多选）（回答完此题后跳至C9）  ①我主动要求做的 ②例行检测时做的，例如征兵、招工、福利体检、献血 ③在接受医疗服务（如手术前）时的检查 ④疾控中心组织做的 ⑤其它（_________________） | （答完此题后跳至C9） | |
| C7 | **如果您没做过，您愿意做免费检测吗？**  ①非常愿意（跳至C9） ②愿意（跳至C9） ③不好说 ④不愿意 ⑤非常不愿意 | （选①、②，跳至C9） | |
| C8 | **如果不愿意，是为什么？**（可多选）  ①觉得自己没有感染艾滋病的风险 ②害怕别人知道自己去做检测 ③不知道在哪里能做该检测 ④不好意思去做艾滋病检测 ⑤不敢面对，害怕检测出自己是艾滋病病毒感染者 ⑥其它（______________） |  | |
| C9 | **您主要是通过哪些途径获得有关艾滋病的知识信息的？**（可多选）  ①网络 ②电视 ③广播 ④医生 ⑤亲戚 ⑥朋友 ⑦报纸杂志 ⑧书籍 ⑨学校教育 ⑩宣传资料 ⑾文艺演出 ⑿宣传广告栏 ⒀咨询服务 ⒁戒毒中心/美沙酮门诊等 ⒂工作组的同伴教育 ⒃不清楚 ⒄其它（请注明_______________） ⒅没有获得过 |  | |
| C10 | **您愿意获得艾滋病防治知识吗？**  ①非常愿意 ②愿意 ③不好说 ④不愿意（跳至D1） ⑤非常不愿意（跳至D1） | （选④、⑤，跳至D1） | |
| C11 | **您希望通过哪些途径获得艾滋病的知识信息？**（可多选）  ①网络 ②电视 ③广播 ④医生 ⑤亲戚 ⑥朋友 ⑦报纸杂志 ⑧书籍 ⑨学校教育 ⑩宣传资料 ⑾文艺演出 ⑿宣传广告栏 ⒀咨询服务 ⒁戒毒中心/美沙酮门诊等 ⒂工作组的同伴教育 ⒃不清楚 ⒄其它（请注明_______________） ⒅不愿意获得 |  | |
| **3、行为特征** | |  | |
| D1 | **您第一次发生性行为是多大年龄？**_________周岁 |  | |
| D2 | **您做这项工作多长时间了？**_______年零_________月 |  |  |
| D3 | **您在这里工作了多长时间了？**_______年零_________月 |  |  |
| D4 | **您目前月收入：**  ①1000元及以下 ②1001~3000元 ③3001~5000元 ④5001~7000元 ⑤ 7001~9000元 ⑥9000元以上 |  | |
| D5 | **您大概多长时间换一次工作地点？** ①不到3个月 ②3个月~ ③6个月~ ④1年~ ⑤从未换过工作 |  | |
| D6 | **通常您一个星期和多少客人发生性行为？** |  | |
| D7 | **您一天之中最多与多少客人发生性行为？** |  | |
| D8 | **最近半年，您与客人发生（阴道交、肛交）性行为时使用安全套的频率？**  ①从未使用（0%） ②偶尔使用（<20%） ③少部分时侯使用（20%~40%） ④半数情况下使用（40%~60%） ⑤大部分时候使用（60%~80%） ⑥经常使用(>80%) ⑦每次都用（100%）（跳至D10） | （选⑦，跳至D10） | |
| D9 | **与客人发生（阴道交、肛交）性行为时没有使用安全套的原因？**（可多选）  ①（当时）没想过用安全套 ②当时身边没有 ③过于昂贵 ④用安全套不舒服 ⑤客人拒绝使用 ⑥客人多付钱让我不用 ⑦害怕对方认为自己不信任他 ⑧不好意思或不敢要求他用 ⑨对方看起来没有（性）病 ⑩采取了其他避孕措施 ⑾采取了其它安全措施（请注明_____） ⑿其它（请注明_____） |  | |
| D10 | **在最近三次与客人发生（阴道交、肛交）性行为时使用了多少次安全套？**  ①一次都没用过 ②1次 ③2次 ④3次 |  | |
| D11 | **最近半年，您与客人发生口交时使用安全套的频率？**  ①从未使用（0%） ②偶尔使用（<20%） ③少部分时侯使用（20%~40%） ④半数情况下使用（40%~60%） ⑤大部分时候使用（60%~80%） ⑥经常使用(>80%) ⑦每次都用（100%）（跳至D13） ⑧未发生口交（跳至D13） | （选⑦、⑧，跳至D13） | |
| D12 | **您与客人发生口交性行为时未使用安全套的原因？**（可多选）  ①（当时）没想过用安全套 ②当时身边没有 ③过于昂贵 ④用安全套不舒服 ⑤客人拒绝使用 ⑥客人多付钱让我不用 ⑦害怕对方认为自己不信任他 ⑧不好意思或不敢要求他用 ⑨对方看起来没有（性）病 ⑩口交不会传播性病/艾滋病 ⑾采取了其它安全措施（请注明_____） ⑿其它（请注明____） |  | |
| D13 | **老板（妈咪）对您们使用安全套是什么态度？** ①规定一定要用 ②没有规定，但提醒您要用 ③老板（妈咪）不管，用不用是您自己的事情 ④不希望你们用 |  | |
| D14 | **如果客人不用安全套，你们不与他们做，老板（妈咪）是否允许？** ①允许 ②不允许 ③不一定 |  | |
| D15 | **最近半年，您与配偶或固定性伴发生性关系的频次？**  ①每周一次及以上 ②每半月一次 ③ 每月一次 ④少于每月一次 ⑤没有发生过（跳至D21） ⑥未婚或无固定性伴（跳至D21） | （选⑤、⑥，跳至D21） | |
| D16 | **最近半年，您与他（配偶或固定性伴）发生（阴道交、肛交）性行为时使用安全套的频率如何？**  ①从未使用（0%） ②偶尔使用（<20%） ③少部分时侯使用（20%~40%） ④半数情况下使用（40%~60%） ⑤大部分时候使用（60%~80%） ⑥经常使用(>80%) ⑦每次都用（100%）（跳至D18） | （选⑦，跳至D18） | |
| D17 | **您与他（配偶或固定性伴）发生（阴道交、肛交）性行为时没有使用安全套的原因？**（可多选）  ①（当时）没想过用安全套 ②当时身边没有 ③过于昂贵 ④用安全套不舒服 ⑤对方拒绝使用 ⑥害怕对方认为自己不信任他 ⑦不好意思或不敢要求他用 ⑧相信对方没病，没必要使用 ⑨害怕对方怀疑我有（性）病 ⑩有生小孩的计划 ⑾采取了其他避孕措施 ⑿采取了其它安全措施（请注明____） ⒀其它（请注明____） |  | |
| D18 | **您最近一次与他（配偶或固定性伴）发生（阴道交、肛交）性行为时使用安全套了吗** ①是 ②否 |  | |
| D19 | **最近半年，您与他（配偶或固定性伴）发生口交时使用安全套的频率？**  ①从未使用（0%） ②偶尔使用（<20%） ③少部分时侯使用（20%~40%） ④半数情况下使用（40%~60%） ⑤大部分时候使用（60%~80%） ⑥经常使用(>80%) ⑦每次都用（100%）（跳至D21） ⑧未发生口交（跳至D21） | （选⑦、⑧，跳至D21） | |
| D20 | **您与他（配偶或固定性伴）发生口交性行为时未使用安全套的原因？**（可多选）  ①（当时）没想过用安全套 ②当时身边没有 ③过于昂贵 ④用安全套不舒服 ⑤对方拒绝使用 ⑥害怕对方认为自己不信任他 ⑦不好意思或不敢要求他用 ⑧相信对方没病，没必要使用 ⑨口交不会传播性病/艾滋病 ⑩采取了其它安全措施（请注明______） ⑾其它（请注明_____） |  | |
| D21 | **最近半年，您有_____个临时性伴。**（若填“0”，跳至D27） | （填“0”，跳至D27） | |
| D22 | **最近半年，您与临时性伴发生（阴道交、肛交）性行为时使用安全套的频率如何？**  ①从未使用（0%） ②偶尔使用（<20%） ③少部分时侯使用（20%~40%） ④半数情况下使用（40%~60%） ⑤大部分时候使用（60%~80%） ⑥经常使用(>80%) ⑦每次都用（100%）（跳至D24） | （选⑦，跳至D24） | |
| D23 | **您与临时性伴发生（阴道交、肛交）性行为时没有使用安全套的原因？**（可多选）  ①（当时）没想过用安全套 ②当时身边没有 ③过于昂贵 ④用安全套不舒服 ⑤对方拒绝使用 ⑥害怕对方认为自己不信任他 ⑦不好意思或不敢要求他用 ⑧相信对方没病，没必要使用 ⑨害怕对方怀疑我有（性）病 ⑩采取了其他避孕措施 ⑾采取了其它安全措施（请注明_______________） ⑿其它（请注明____________） |  | |
| D24 | **您最近一次与临时性伴发生（阴道交、肛交）性关系时使用安全套了吗？** ①是 ②否 |  | |
| D25 | **最近半年，您与临时性伴发生口交时使用安全套的频率？**  ①从未使用（0%） ②偶尔使用（<20%） ③少部分时侯使用（20%~40%） ④半数情况下使用（40%~60%） ⑤大部分时候使用（60%~80%） ⑥经常使用(>80%) ⑦每次都用（100%）（跳至D27） ⑧未发生口交（跳至D27） | （选⑦、⑧，跳至D27） | |
| D26 | **您与临时性伴发生口交性行为时未使用安全套的原因？**（可多选）  ①（当时）没想过用安全套 ②当时身边没有 ③过于昂贵 ④用安全套不舒服 ⑤对方拒绝使用 ⑥害怕对方认为自己不信任他 ⑦不好意思或不敢要求他用 ⑧相信对方没病，没必要使用 ⑨口交不会传播性病/艾滋病 ⑩采取了其它安全措施（请注明___________） ⑾其它（请注明________） |  | |
| D27 | **您与他人发生性行为使用安全套时是否是全程使用？**  ①每次均未全程使用（0%） ②偶尔全程使用（<20%） ③少部分时侯全程使用（20%~40%） ④半数情况下全程使用（40%~60%） ⑤大部分时候全程使用（60%~80%） ⑥经常全程使用(>80%) ⑦每次都全程使用（100%）（跳至D29） | （选⑦，跳至D29） | |
| D28 | **您与他人发生性行为时没有全程使用安全套的原因？**（可多选）  ①只是用于避孕，在射精前才使用 ②有时安全套滞留于阴道中 ③只有精液才会传播性病、艾滋病，只在射精前使用 ④相信配偶没病，对配偶没有全程使用 ⑤相信临时性伴没病，对临时性伴没有全程使用 ⑥相信熟客没病，对熟客没有全程使用 ⑦客人看起来没病 ⑧对方不愿意全程使用 ⑨其它（请注明_____________） |  | |
| D29 | **您知道的与您发生过性关系的人是否有查出是艾滋病阳性者的？**  ①有 ②没有 ③不能确定 |  | |
| D30 | **您是否出现过下列性病症状？**（排尿痛或烧灼感；（男性）尿道分泌物异常/（女性）阴道分泌物异常；生殖器出现皮肤破损、溃疡或增生物；性交疼痛；严重或持续的盆腔疼痛）  ①出现过 ②未出现过（跳至D32） | （选②，跳至D32） | |
| D31 | **您出现（性病）症状一般是如何解决的？**  ①到性病专科门诊就诊 ②到区县级以上综合医院就诊 ③妇幼保健院就诊 ④到私人诊所就诊 ⑤乡镇卫生院（社区卫生服务中心）就诊 ⑥村卫生室（社区卫生服务站）就诊 ⑦自己买药治疗 ⑧不作处理 ⑨其它（请注明____________） |  | |
| D32 | **您是否曾被医生诊断过得了性病？** ①是 ②否（跳至D35） | （选②，跳至D35） | |
| D33 | **医生告诉您得的是什么病？** （可多选）  ①梅毒 ②生殖器疱疹 ③淋病 ④尖锐湿疣 ⑤非特异性尿道炎 ⑥其它（请注明_____） ⑦不清楚 |  | |
| D34 | **最近一次得性病是何时?** ①一年之内 ②一年以前 ③三年以前 ④五年以前 |  | |
| D35 | **最近半年，您饮酒的频率怎样？**  ①基本上每天都喝 ②至少3次/周 ③至少1次/周 ④每周少于1次 ⑤从不饮酒（跳至D37） | （选⑤，跳至D37） | |
| D36 | **您和性伴发生性行为之前，使用酒精的情况怎样？**  ①从未使用（0%） ②偶尔使用（<20%） ③少部分时侯使用（20%~40%） ④半数情况下使用（40%~60%） ⑤大部分时候使用（60%~80%） ⑥经常使用(>80%) ⑦每次都用（100%） |  | |
| D37 | **您最近半年内使用过下列哪些物质？（可多选）**  ①未使用（跳至D42） ②摇头丸 ③冰毒 ④K粉（氯胺酮） ⑤鸦片 ⑥大麻 ⑦海洛因 ⑧麻古 ⑨杜冷丁 (10)吗啡 (11)其它（请注明_______________________） | （选①，跳至D42） | |
| D38 | **您最近半年是否在性行为前使用过上述物质？**  ①是 ②否（跳至D40） | （选②，跳至D40） | |
| D39 | **最近半年您和性伴发生性行为之前，服用上述药物的情况怎样？**  ①从未使用（0%） ②偶尔使用（<20%） ③少部分时侯使用（20%~40%） ④半数情况下使用（40%~60%） ⑤大部分时候使用（60%~80%） ⑥经常使用(>80%) ⑦每次都用（100%） |  | |
| D40 | **您最近半年是否注射过以上物质？** ①是 ②否（跳至D42） | （选②，跳至D42） | |
| D41 | **您知道与您共用过针具者是否现在有查出是艾滋病阳性者的？**①有 ②没有 ③不能确定 ④未共用过针具 |  | |
| D42 | **您认为自己感染艾滋病的可能性怎样？** ①非常大 ②大 ③一般 ④小 ⑤非常小 |  | |
| D43 | **您认为自己感染艾滋病的危险主要来自：_______**（多选，并排序）  ①陌生客人 ②熟客 ③临时性伴 ④配偶/固定性伴 ⑤其他（请注明_______________） |  | |

**表3. 对艾滋病预防措施的知识、态度、**使用意愿

| **序号** | **问题及选项** | | | | **回答** | | | |  |
| --- | --- | --- | --- | --- | --- | --- | --- | --- | --- |
| E1 | **就目前而言，您认为预防艾滋病困难吗？** ①非常困难 ②困难 ③一般 ④不困难 ⑤完全不困难 | | | |  | | | |  |
| E2 | **您认为自己有能力预防艾滋病感染吗？**  ①完全有能力 ②有能力 ③不好说 ④没有能力 ⑤完全没有能力 | | | |  | | | |  |
| E3 | **您是否同意下列说法？** | | | |  | | | |  |
|  | 1. **很少有男人喜欢用安全套**   ①完全同意 ②基本同意 ③不好说 ④基本不同意 ⑤完全不同意 | | | |  | | | |  |
|  | 1. **如果性伴没有提出，您会主动提出使用安全套**   ①完全同意 ②基本同意 ③不好说 ④基本不同意 ⑤完全不同意 | | | |  | | | |  |
|  | 1. **如果您坚持使用安全套，其他人会认为您得了性病**   ①完全同意 ②基本同意 ③不好说 ④基本不同意 ⑤完全不同意 | | | |  | | | |  |
|  | 1. **如果对方不愿意使用安全套，您能说服他使用安全套**   ①完全同意 ②基本同意 ③不好说 ④基本不同意 ⑤完全不同意 | | | |  | | | |  |
|  | 1. **如果对方拒绝用安全套的话，您会拒绝与他发生性行为**   ①完全同意 ②基本同意 ③不好说 ④基本不同意 ⑤完全不同意 | | | |  | | | |  |
|  | 1. **您知道如何正确使用安全套**   ①完全同意 ②基本同意 ③不好说 ④基本不同意 ⑤完全不同意 | | | |  | | | |  |
|  | 1. **如果您坚持用安全套，客人会生气**   ①完全同意 ②基本同意 ③不好说 ④基本不同意 ⑤完全不同意 | | | |  | | | |  |
|  | 1. **如果您坚持用安全套，客人就不找您了**   ①完全同意 ②基本同意 ③不好说 ④基本不同意 ⑤完全不同意 | | | |  | | | |  |
|  | 1. **不用安全套，客人会多付钱**   ①完全同意 ②基本同意 ③不好说 ④基本不同意 ⑤完全不同意 | | | |  | | | |  |
|  | 1. **不用安全套，省时间省钱**   ①完全同意 ②基本同意 ③不好说 ④基本不同意 ⑤完全不同意 | | | |  | | | |  |
|  | 1. **通过使用安全套可以保护自己不得性病、艾滋病**   ①完全同意 ②基本同意 ③不好说 ④基本不同意 ⑤完全不同意 | | | |  | | | |  |
| E4 | **您以前听说过下列预防艾滋病的措施吗？您认为它可能的预防效果怎样（调查员解释下列措施）** | | | |  | | | |  |
|  | **（1）阴道/直肠杀微生物剂**  ①听说过 ②未听说过 | | | |  | | | |  |
|  | **您认为它可能的预防效果**：①非常好 ②好 ③一般 ④差 ⑤非常差 | | | |  | | | |  |
|  | **（2）艾滋病暴露后用药** ①听说过 ②未听说过 | | | |  | | | |  |
|  | **您认为它可能的预防效果**：①非常好 ②好 ③一般 ④差 ⑤非常差 | | | |  | | | |  |
|  | **（3）艾滋病暴露前用药** ①听说过 ②未听说过 | | | |  | | | |  |
|  | **您认为它可能的预防效果**：①非常好 ②好 ③一般 ④差 ⑤非常差 | | | |  | | | |  |
| E5 | **您使用药物预防过性病吗？** ①用过 ②未用过（跳至E10） | | | | （选②，跳至E10） | | | |  |
| E6 | **如果您使用过，是何种药物？** | | | |  | | | |  |
| E7 | **药物的用法是：** ①口服 ②外用 ③注射 | | | |  | | | |  |
| E8 | **是谁建议/给您使用的？**（可多选）  ①医生 ②性伴 ③朋友 ④其它（请注明________）⑤没有人，自己使用的 | | | |  | | | |  |
| E9 | **具体的使用时间是：** （可多选）  ①高危行为发生前 ②高危行为发生后 ③（多次）发生高危行为期间有规律的使用 | | | |  | | | |  |
| E10 | **您使用过药物预防艾滋病吗？** ①用过 ②未用过 （跳至E15） | | | | （选②，跳至E15） | | | |  |
| E11 | **如果您使用过，是何种药物？** | | | |  | | | |  |
| E12 | **药物的用法是：** ①口服 ②外用 ③注射 | | | |  | | | |  |
| E13 | **是谁建议/给您使用的？**（可多选）  ①医生 ②性伴 ③朋友 ④其它（请注明________）⑤没有人，自己使用的 | | | |  | | | |  |
| E14 | **具体的使用时间是：** （可多选）  ①高危行为发生前 ②高危行为发生后 ③（多次）发生高危行为期间有规律的使用 | | | |  | | | |  |
| E15 | **您知道您周围有人使用过药物预防艾滋病吗？** ①有 ②无（跳至E19） | | | | （选②，跳至E19） | | | |  |
| E16 | **如果您周围有人使用过，是何种药物？** | | | |  | | | |  |
| E17 | **药物的用法是：** ①口服 ②外用 ③注射 | | | |  | | | |  |
| E18 | **具体的使用时间是：** （可多选）  ①高危行为发生前 ②高危行为发生后 ③（多次）发生高危行为期间有规律的使用 ④不清楚 | | | |  | | | |  |
| E19 | **假如您与配偶/固定性伴发生性关系时不采取预防措施，您认为您感染艾滋病的机会如何？**  ①非常大 ②大 ③一般 ④小 ⑤非常小 ⑥无配偶/固定性伴 | | | |  | | | |  |
| E20 | **假如您与客人发生性关系时不采取预防措施，您认为您感染艾滋病的机会如何？**  ①非常大 ②大 ③一般 ④小 ⑤非常小 | | | |  | | | |  |
| E21 | **假如您与临时性伴发生性关系时不采取预防措施，您认为您感染艾滋病的机会如何？**  ①非常大 ②大 ③一般 ④小 ⑤非常小 ⑥无临时性伴 | | | |  | | | |  |
| E22 | **如果证明艾滋病暴露前用药对预防艾滋病安全有效，您认为是否应该在性工作者中推广这种药物？** ①完全应该 ②应该 ③不好说 ④不应该 ⑤完全不应该 | | | |  | | | |  |
| E23 | **下面是您对艾滋病暴露前用药的使用意愿** | | | |  | | | |  |
|  | **（1）如果艾滋病暴露前用药对预防艾滋病安全、有效，您会使用吗？**  ①肯定会（跳至E25） ②很可能会（跳至E25） ③不太清楚 ④很可能不会 ⑤肯定不会 | | | | （选①、②，跳至E25） | | | |  |
|  | **（2）如果艾滋病暴露前用药对预防艾滋病安全、有效，且免费提供，您会使用吗？**  ①肯定会（跳至E25） ②很可能会（跳至E25） ③不太清楚 ④很可能不会 ⑤肯定不会 | | | | （选①、②，跳至E25） | | | |  |
|  | **（3）如果艾滋病暴露前用药对预防艾滋病安全、有效，免费提供，且您周围有少数人在服用这种药物，您会使用吗？**①肯定会（跳至E25） ②很可能会（跳至E25）） ③不太清楚 ④很可能不会 ⑤肯定不会 | | | | （选①、②，跳至E25） | | | |  |
|  | **（4）如果艾滋病暴露前用药对预防艾滋病安全、有效，免费提供，且您周围有较多人在服用这种药物，您会使用吗？**①肯定会（跳至E25） ②很可能会（跳至E25） ③不太清楚 ④很可能不会 ⑤肯定不会 | | | | （选①、②，跳至E25） | | | |  |
| E24 | **您（可能）不愿意服用艾滋病暴露前用药的原因？**（可多选）  ①认为自己没有感染艾滋病的风险 ②担心药物的副作用 ③担心他人的歧视 ④担心家人、朋友的反对 ⑤担心客人的反对，影响生意 ⑥担心老板（妈咪）的反对 ⑦怀疑药物的效果 ⑧其它（请注明_____________） | | | |  | | | |  |
| E25 | **如果艾滋病暴露前用药对预防艾滋病安全、有效，您会建议您朋友使用吗？**  ①肯定会 ②很可能会 ③不太清楚 ④很可能不会 ⑤肯定不会 | | | |  | | | |  |
| **若不愿意服用艾滋病暴露前用药（E23(4)选择④或⑤），则结束此部分问卷，跳转至心理人格量表** | | | | |  | | | | |
| E26 | **对于艾滋病暴露前用药，您关注什么？请按照您最关心的方面进行排序。**（多选，并排序）  ①效果（有效性） ②安全性（副作用） ③费用 ④获得的方便性 ⑤服用的方便性 ⑥周围人的看法 ⑦性伙伴的支持 ⑧家人的支持 ⑨周围人是否使用 ⑩其它（请注明______________） ⑾我根本不关心 | | | |  | | | |  |
| E27 | **您担心服用药物时被他人发现吗？担心被哪些人发现？**（可多选）  ①不担心被人发现 ②客人 ③配偶/固定性伴 ④临时性伴 ⑤其他家人 ⑥朋友 ⑦老板（妈咪）⑧其他人 | | | |  | | | |  |
|  |  | 配偶/固定 | 临时性伴 | 客人 | 其他家人 | 朋友 | 老板 | |  |
| E28 | **您认为下列人员对于您用药的态度怎样？**  ①支持 ②不太清楚 ③反对 ④无此种关系的人 |  |  |  |  |  |  | |  |
|  |  | （选④，跳至下一个） （选④，跳至下一个） | | | | | |  | |
| E29 | **您认为下列人员的态度会影响您的选择吗？**  ①肯定会 ②很可能会 ③不太清楚 ④很可能不会 ⑤肯定不会 |  |  |  |  |  |  | |  |
| E30 | **如果客人知道您在服用这种药物，您认为您的客人会怎样？**（可多选）  ①生气 ②不再和您发生性关系 ③更有安全感 ④以为您得了病 ⑤要求您不用 ⑥其它（请注明_______） | | | |  | | | |  |
| E31 | **下列说法，您是否同意？** | | | |  | | | |  |
|  | 1. **如果他人知道您在服用这种药物，他/她们会歧视您**   ①完全同意 ②基本同意 ③不好说 ④基本不同意 ⑤完全不同意 | | | |  | | | |  |
|  | 1. **无论其他人怎么看，我都相信我能坚持服用这种药物**   ①完全同意 ②基本同意 ③不好说 ④基本不同意 ⑤完全不同意 | | | |  | | | |  |
| E32 | **如果艾滋病暴露前用药需要自己花钱，您最多愿意每月花多少钱。**  ①不愿意花钱 ②<100元 ③100～元 ④200～元 ⑤400～元 ⑥600～元 ⑦800～元 ⑧>1000元 | | | |  | | | |  |
| E33 | **如果这种药物需要每天服用，您会坚持服用这种药物吗？**  ①肯定会（跳至E35） ②很可能会（跳至E35） ③不太清楚 ④很可能不会 ⑤肯定不会 | | | | （选①、②，跳至E35） | | | |  |
| E34 | **如果您不愿意每天服用，您能忍受的最短服药周期为：**  ①隔天一次 ②三天一次 ③每周一次 ④半月一次 ⑤一月一次 ⑥其它（请注明_________） | | | |  | | | |  |
| E35 | **如果有两种类型的药，第一种药价格便宜，但服药间隔期短，第二种药价格较贵，但服药间隔期长，您更倾向于选择哪种？**①第一种 ②第二种 ③不知道 | | | |  | | | |  |
| E36 | **您希望通过哪种途径了解艾滋病暴露前用药的信息？**（可多选）  ①网络 ②电视 ③广播 ④医生 ⑤亲戚 ⑥朋友 ⑦报纸杂志 ⑧书籍 ⑨学校教育 ⑩宣传资料 ⑾文艺演出 ⑿宣传广告栏 ⒀咨询服务 ⒁戒毒中心/美沙酮门诊等 ⒂工作组的同伴教育 ⒃不清楚 ⒄其它（请注明_______________） ⒅不愿意获得 | | | |  | | | |  |
| E37 | **您希望在哪里获得艾滋病暴露前用药？** （可多选）  ①村卫生室/卫生服务站 ②乡镇卫生院/卫生服务中心 ③县（区）级以上医院 ④疾控中心（卫生防疫站） ⑤艾滋病咨询机构 ⑥性病专科门诊 ⑦戒毒所/美沙酮门诊 ⑧私人诊所 ⑨药店 ⑩成人保健用品商店 ⑾其它（请注明）________________ | | | |  | | | |  |
| E38 | **您选择在该地点获得艾滋病暴露前用药的原因是：**（可多选）  ①保密性强 ②方便 ③该机构可靠，可信赖 ④其它（请注明_________________） | | | |  | | | |  |
| E39 | **如果使用暴露前用药，您认为您在与他人发生性关系时使用安全套的频率会怎样？**  ①肯定上升 ②很可能上升 ③与以前一样 ④很可能下降 ⑤肯定下降 | | | |  | | | |  |
| E40 | **如果使用暴露前用药，您认为您的性伙伴个数会怎样？**  ①肯定上升 ②很可能上升 ③与以前一样 ④很可能下降 ⑤肯定下降 | | | |  | | | |  |
| E41 | **您愿意参加艾滋病暴露前用药的临床试验吗？**  ①非常愿意（仅回答E42题后跳至心理人格量表） ②愿意（仅回答E42题后跳至心理人格量表） ③不好说（既要回答E42题，又要回答E43题） ④不愿意（跳至E43） ⑤非常不愿意（跳至E43） | | | | （选④、⑤，跳至E43） | | | |  |
| E42 | **您（可能）愿意参加艾滋病暴露前用药试验的原因是：**（可多选）  ①担心自己有患艾滋病的危险 ②希望能够推广一种艾滋病暴露前预防用药 ③参加试验不用花钱，可以免费服用预防药物 ④其它（请注明________________________） | | | |  | | | |  |
| E43 | **您（可能）不愿意参加艾滋病暴露前用药试验的原因是：**（可多选）  ①自己没有患艾滋病的危险，没有必要参加 ②担心药物的副作用 ③担心药物没有效果 ④担心自己服用药物会受到他人的歧视 ⑤担心性伙伴反对 ⑥担心家人反对 ⑦其他（请注明_______） | | | |  | | | |  |
